# Getting back to nature: a reality check for experiments in controlled environments

**DOI:** 10.1093/jxb/erx220

**Published:** 2017-06-29

**Authors:** Maria Grazia Annunziata, Federico Apelt, Petronia Carillo, Ursula Krause, Regina Feil, Virginie Mengin, Martin A Lauxmann, Karin Köhl, Zoran Nikoloski, Mark Stitt, John E Lunn

**Affiliations:** 1 Max Planck Institute of Molecular Plant Physiology, Am Mühlenberg, Potsdam-Golm, Germany; 2 University of Campania “Luigi Vanvitelli”, Via Vivaldi, Caserta, Italy; 3 University of Potsdam, Karl-Liebknecht-Str., Potsdam, Germany

**Keywords:** Amino acid, Arabidopsis, thaliana, controlled environment, LED lighting, visible light spectrum, organic acid, starch, sucrose, trehalose 6-phosphate

## Abstract

Irradiance from sunlight changes in a sinusoidal manner during the day, with irregular fluctuations due to clouds, and light–dark shifts at dawn and dusk are gradual. Experiments in controlled environments typically expose plants to constant irradiance during the day and abrupt light–dark transitions. To compare the effects on metabolism of sunlight versus artificial light regimes, *Arabidopsis thaliana* plants were grown in a naturally illuminated greenhouse around the vernal equinox, and in controlled environment chambers with a 12-h photoperiod and either constant or sinusoidal light profiles, using either white fluorescent tubes or light-emitting diodes (LEDs) tuned to a sunlight-like spectrum as the light source. Rosettes were sampled throughout a 24-h diurnal cycle for metabolite analysis. The diurnal metabolite profiles revealed that carbon and nitrogen metabolism differed significantly between sunlight and artificial light conditions. The variability of sunlight within and between days could be a factor underlying these differences. Pairwise comparisons of the artificial light sources (fluorescent versus LED) or the light profiles (constant versus sinusoidal) showed much smaller differences. The data indicate that energy-efficient LED lighting is an acceptable alternative to fluorescent lights, but results obtained from plants grown with either type of artificial lighting might not be representative of natural conditions.

## Teaser

The carbon and nitrogen metabolism of Arabidopsis plants grown in sunlight differs from plants grown with artificial light, even when the spectral quality and sinusoidal profile of sunlight are approximated experimentally.

## Introduction

Plants growing in natural environments experience diurnal fluctuations in irradiance and light quality, with gradual shifts between light and dark at dawn and dusk (Vialet-Chabrand *et al.*, 2016, 2017). In contrast, experiments in controlled environment chambers typically expose plants to a constant irradiance during the day, and abrupt transitions between light and dark at dawn and dusk. Growth chambers do offer some advantages: the experiments are easily reproducible and allow responses to a single factor (e.g. day length, irradiance, temperature) to be investigated (Jänkänpää *et al.*, 2012; Poorter *et al.*, 2016). Abrupt light–dark transitions can also be informative, as they can give insights into the plant’s response to increasing or decreasing irradiance by exaggerating those responses, making them easier to observe experimentally (Fondy *et al.*, 1989; Gibon *et al.*, 2004; Graf *et al.*, 2010). Nevertheless, plants grown in controlled environments can show significantly different phenotypes from plants of the same genotype grown in more natural conditions or in the field (Servaites *et al.*, 1989; Geiger *et al.*, 1991; Bruce *et al.*, 2015; Vialet-Chabrand *et al.*, 2016, 2017). However, there is a paucity of information about which of the parameters that differentiate natural and artificial environments have the greatest impact on plant metabolism and growth.

The light in controlled environment chambers can differ in several ways from natural sunlight. In chambers, plants are usually grown with a constant (square-wave) irradiance during the day, whereas sunlight changes in a sinusoidal manner, peaking at noon with no abrupt transitions at dawn and dusk, and often fluctuates due to changes in cloud cover or shading. Although sinusoidal light profiles can be simulated in controlled environment chambers, such conditions appear to be rarely used, and even fewer studies have directly compared sinusoidal and square-wave conditions. One of the few examples is a series of experiments on sugar beet (*Beta vulgaris*), which showed that the more gradual increase in irradiance in the sinusoidal regime led to sequential activation of photosynthetic enzymes, resulting in a more efficient carbon flow through the Calvin–Benson cycle into starch and sucrose (Fondy *et al.*, 1989; Servaites *et al.*, 1989).

Another important difference between artificial and natural light regimes is spectral quality. Controlled environment chambers operated with low-medium light levels (up to about 300 µmol m^−2^ s^−1^) are typically illuminated with cool-white fluorescent tubes, sometimes supplemented with red-enhanced fluorescent tubes or incandescent light bulbs. High-pressure discharge lamps (e.g. mercury, sodium, or metal halide lamps) are used to achieve higher light levels. All of these types of artificial lighting produce light spectra with very uneven wavelength distributions, with sharp peaks around the main emission wavelengths and little or no light in between. In contrast, the visible light spectrum of sunlight is much more homogeneous. The spectral quality of the light used in controlled environment experiments is often given little attention in plant metabolic studies, even though it can have a significant impact on photosynthetic metabolism. For example, in a study of photoassimilate partitioning in pangola grass (*Digitaria eriantha*) plants grown under different types of artificial lighting, it was observed that plants grown under ‘full spectrum’ fluorescent tubes accumulated more starch during the day than plants grown with cool-white fluorescent tubes, but less than plants grown under low-pressure sodium lamps (Britz *et al.*, 1985).

Recent technical advances in the development of light emitting diodes (LEDs) have increased their spectral range and luminosity, making this type of lighting a potential alternative to fluorescent tubes as a source of illumination in controlled environment chambers (Köhl *et al.*, 2017). By combining two or more different types of LED, it is also possible to generate emission spectra that are more homogeneous than those from other types of artificial lighting, and which can be tuned to produce spectra that simulate sunlight more closely. With their lower energy consumption, lower operating temperature, and longer life, LEDs also offer economic and environmental benefits compared to other types of artificial lighting.

In this study, we investigated the effect of different natural and artificial light regimes on the metabolic phenotypes of arabidopsis (*Arabidopsis thaliana*) plants when other environmental parameters were held constant.

## Materials and methods

### Plant material and growth conditions

*Arabidopsis thaliana* [L.] Heynh. accession Columbia 0 from the MPI-MP in-house collection was used in all experiments. Seeds were sown on soil mixed with vermiculite (1:1) and grown in a controlled environment chamber for 7 d with a 16-h photoperiod and irradiance of 250 µmol m^−2^ s^−1^, day/night temperatures of 20/6 °C, and a constant 75% relative humidity (RH). Plants were then transferred to an 8-h photoperiod with an irradiance of 100 µmol m^−2^ s^−1^, day/night temperatures of 20/16 °C and 60/75% RH. At 14 d after sowing plants were transplanted to 10-cm-diameter pots (five plants per pot) of soil:vermiculite (1:1) and transferred to either a naturally illuminated greenhouse or a controlled environment chamber. The greenhouse experiments were conducted in Potsdam, Germany (52°24′55.2′′N, 12°58′5.5′′E) around the vernal equinox in March 2012 and March 2015. Due to differences in cloud cover, the daily light integrals (DLIs) were 7 mol m^−2^ d^−1^ and 12 mol m^−2^ d^−1^, respectively. The irradiance profiles on the day of harvest and preceding days are shown in Supplementary Figs S1 and S2, respectively, at *JXB* online. In both years the temperature range in the greenhouse was maintained between 22–24 °C during the day and 18–22 °C at night, and RH was 60%.

Four different light regimes were used for experiments in controlled environment chambers, each with a 12-h photoperiod (Table 1), as follows.

**Table 1.** Overview of light regimes

Light regime	Light source	Diurnal profile	Photoperiod (h)	DLI (mol m^–2^d^–1^)
1	Sunlight	Sinusoidal	12^a^	7^a^
2	Fluorescent	Square	12	7
3	Fluorescent	Sinusoidal	12	7
4	LED	Square	12	7
5	LED	Sinusoidal	12	7
6	Sunlight	Sinusoidal	12^b^	12^b^
7	Fluorescent	Sinusoidal	12	12

^a^ Vernal equinox 2012; ^b^ Vernal equinox 2015.

(i) Fluorescent, square-wave. Plants were grown in a Percival Scientific AR-75L cabinet (www.percival-scientific.com) with white fluorescent tubes giving a constant irradiance of 160 µmol m^−2^ s^−1^ (DLI=7 mol m^−2^ d^−1^). Day/night temperatures were 20/18 °C, and RH was 65–75%.

(ii) Fluorescent, sinusoidal. Plants were grown in a Percival Scientific E-36 L cabinet (supplied by CLF Plant Climatics, Wertingen, Germany; http://www.plantclimatics.de) with variable fluorescent lighting operated in sinusoidal mode with midday peak irradiance of 235 µmol m^−2^ s^−1^ (DLI=7 mol m^−2^ d^−1^) or 455 µmol m^−2^ s^−1^ (DLI=12 mol m^−2^ d^−1^). Day/night temperatures were 20/18 °C and RH was 65–75%.

(iii) LED, square-wave. Plants were grown in a custom-built phytotron (Johnson Controls, Mannheim, Germany; http://www.johnsoncontrols.com) and illuminated with a mobile R-MU-1002 LED system (RHENAC GreenTec AG, Hennef, Germany; http://www.rhenac-greentec.de/) with a constant irradiance of 160 µmol m^−2^ s^−1^ (DLI=7 mol m^−2^ d^−1^). The LEDs were tuned to give a spectrum based on standard illuminant D65 (as defined by the International Commission on Illumination), which approximates the midday spectrum of sunlight in northern/western Europe (Supplementary Fig. S3). Day/night temperatures were 20/18 °C, and RH was 60–75% RH.

(iv) LED, sinusoidal. Plants were grown as in (iii) but with a sinusoidal light profile and a peak irradiance of 250 µmol m^−2^ s^−1^ (DLI=7 mol m^−2^ d^−1^). The light spectra produced by the fluorescent and LED lights are compared with the spectrum of sunlight in Supplementary Fig. S4. In comparison with the highly heterogeneous spectrum of white fluorescent lights, the LED array gave a more homogeneous distribution of wavelengths that was more similar to sunlight, but the LED spectrum had more pronounced peaks in the red and blue regions of the spectrum than sunlight. In all controlled environment experiments, the position of the pots within the chamber was rotated on a daily basis to minimize positional effects.

In each growth condition, 4-week-old plants were harvested through a 24-h light–dark cycle. Sampling was performed at 30-min or 1-h intervals around dawn and dusk, and at 2-h intervals during the rest of the diurnal cycle. Rosettes were harvested in the ambient light conditions and immediately frozen in liquid nitrogen. Rosettes from five plants were pooled for each biological replicate. At each time point the harvest was completed within 5 min. The frozen tissue was ground to a fine powder at –70 °C using an automated cryogenic grinding robot (Stitt *et al.*, 2007) and stored at –80 °C until analysis.

### Metabolite and enzyme assays

Soluble sugars (glucose, fructose, and sucrose) were extracted with boiling 80% (v/v) ethanol and assayed enzymatically as described by Stitt *et al.* (1989). Starch was determined enzymatically in the insoluble material remaining after the ethanolic extraction (Hendriks *et al.*, 2003). Trehalose 6-phosphate (Tre6P), phosphorylated intermediates, and organic acids were extracted with chloroform/methanol, and measured by high-performance anion-exchange liquid chromatography coupled to tandem mass spectrometry (LC-MS/MS) as described by Lunn *et al.* (2006), with modifications as described in Figueroa *et al.* (2016). Amino acids were extracted from 50 mg of powdered material by mixing with 1 mL of 40% (v/v) ethanol. After extraction at –20 °C for 24 h, the mixture was centrifuged at 14 000 *g*, 4 °C, for 10 min. Primary amino acids were measured in the supernatant by HPLC with fluorescent detection following pre-column derivatization with *O*-phthaldialdehyde as described by Carillo *et al.* (2011). Proline was determined colorimetrically using ninhydrin as described by Woodrow *et al.* (2017).

### Statistical analysis

Statistical analyses of the data were performed using the pairwise Student’s *t*-test function of SigmaPlot 12.5 (Systat Software GmbH, Erkrath, Germany; www.systat.de). Principal component analysis (PCA) was performed using XLStat-Base software (Addinsoft, New York, USA; www.xlstat.com). The RV coefficient, originally introduced by Escoufier (1973), is a measure of similarity between two positive semi-definite matrices (e.g. covariance matrices) obtained from the same set of variables. Given two covariance matrices, A and B, the RV coefficient is defined as:

$$\text{RV}=\text{tr}\left(A^{T}B\right)/{\left[\text{tr}\left(A^{T}A\right)\text{tr}\left(B^{T}B\right)\right]}^{1/2}$$

where T denotes the matrix transpose and tr is the trace of the matrix.

## Results

### Global comparisons of plants grown under natural and artificial light at DLI 7

**Fig. 1.**
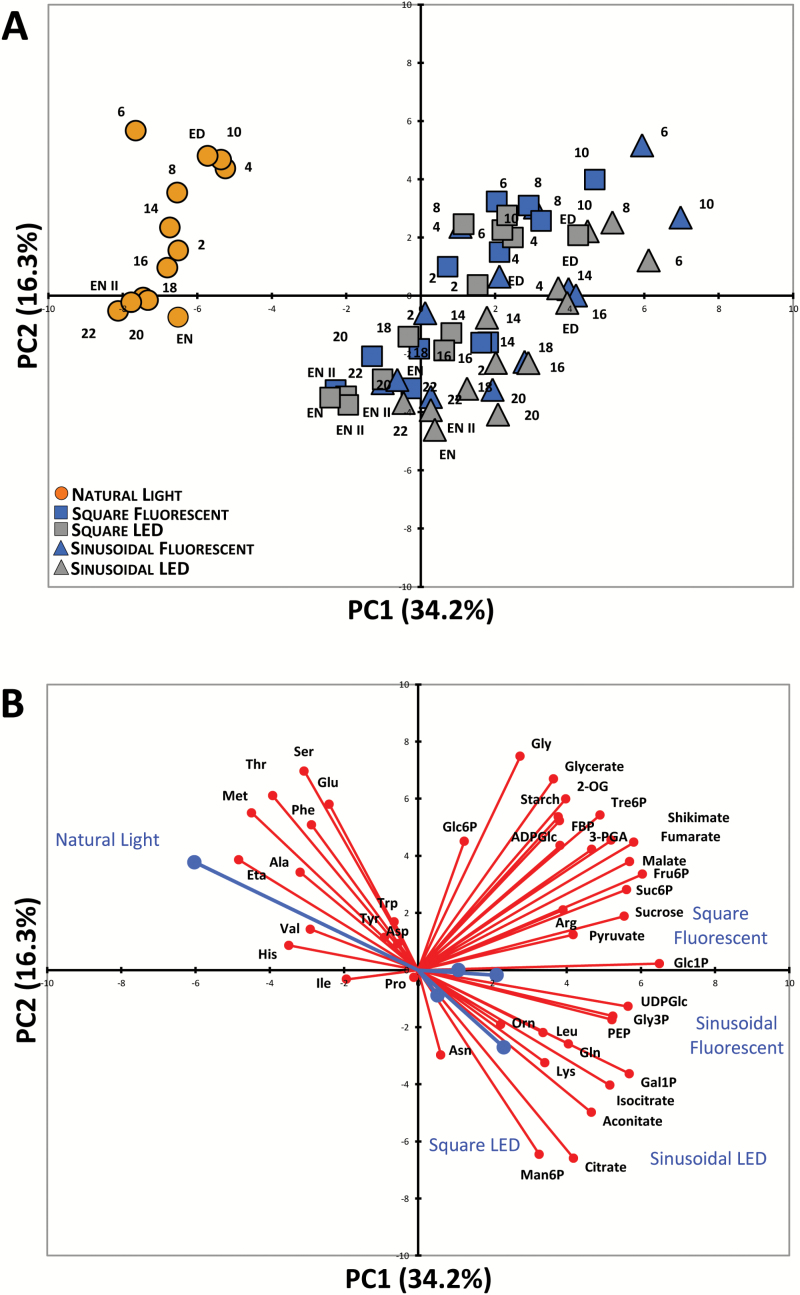
Principal component analysis (PCA) of metabolite data from Arabidopsis plants. (A) PCA of metabolite data from plants grown in a naturally illuminated greenhouse (orange circles) or in controlled environment chambers with a 12-h photoperiod and daily light integral (DLI) of 7 mol m^−2^ d^−1^. The artificial illumination was provided by white fluorescent tubes (blue symbols) or LED lights (grey symbols), with either a constant (squares) or sinusoidal (triangles) light profile during the day. Numbers indicate the time of harvest in hours after dawn (zeitgeber time, ZT); ED, end of day (ZT12); EN I, end of preceding night (ZT0); EN II, end of night (ZT24). The percentages of total variance represented by principal component 1 (PC1) and principal component 2 (PC2) are shown in parentheses. (B) The loadings of individual metabolites (red) on the principal components shown in (A) and the (average) loadings of the individual experimental conditions (blue). Glucose and fructose were not included in the PCA due to the very high variability in the data.

Plants were grown in five different light regimes with a 12-h photoperiod and DLI of 7 mol m^−2^ d^−1^ (Table 1), and harvested at 0.5- to 2-h intervals during a complete 24-h light–dark cycle for metabolite analysis. Representative plants from each light regime are shown in Supplementary Fig. S5 from the day of harvest. All of the plants had a similar rosette diameter and developmental stage (approx. 10–12 leaves and non-flowering), indicating only minor differences in growth rates between conditions. We used PCA to get an initial overview of the data (Fig. 1). Along the principal component 1 (PC1) axis, we identified a clear separation of the samples from natural-light grown plants versus those grown under artificial lighting. The latter clustered together with no obvious separation between fluorescent versus LED, or sinusoidal versus square light regimes (Fig. 1A). Along the PC2 axis there was a separation of samples harvested in the light (ZT0–ZT12) versus the dark (ZT12–ZT24).

The separation between plants grown under natural and artificial light along PC1 was driven mainly by differences in sucrose, phosphorylated intermediates, and organic acids (all positive loadings) and amino acids (Fig. 1B). Except for Gln, Arg, Lys, Leu, Gly, and ornithine, which had positive loadings, the rest of the amino acids had large negative loadings. Separation between light- and dark-harvested samples along the PC2 axis was also driven in part by many of the same amino acids (although the directionality of the loadings was reversed), along with a strong influence from organic acids, starch, and some phosphorylated intermediates.

To interrogate the data from plants grown in artificial light in more detail, data from sinusoidal versus square light regimes were compared separately for plants grown under fluorescent (see Supplementary Fig. S6A, B) or LED lighting (Supplementary Fig. S6C, D). For both types of lighting the primary separation along the PC1 axes was between light- and dark-harvested samples, but there was a clear separation between the two light regimes along the PC2 axes. The reciprocal comparisons were also done, comparing fluorescent versus LED lighting for plants grown with a sinusoidal (Supplementary Fig. S7A, B) or square light profile (Supplementary Fig. S7C, D). For both light regimes, the primary separation along the PC1 axes was also between light- and dark-harvested samples, with both datasets showing similar trajectories through the light–dark cycle, but there was again a clear separation between the two different types of lighting along the PC2 axes. The metabolites contributing to these separations are indicated by the loading vectors in Supplementary Figs S6B, D, S7B, D. The separation of light- and dark-harvested samples (PC1) was driven mainly by differences in organic acids and phosphorylated intermediates. Amino acids were prominent among the metabolites contributing to the separations between light regimes (PC2), with tricarboxylic acids (citrate, aconitate, and isocitrate) and some hexose-phosphates (Glc6P, Man6P) also being important in several of the pairwise comparisons.

### Carbon and nitrogen metabolism in plants grown under natural versus artificial light at DLI 7

**Fig. 2.**
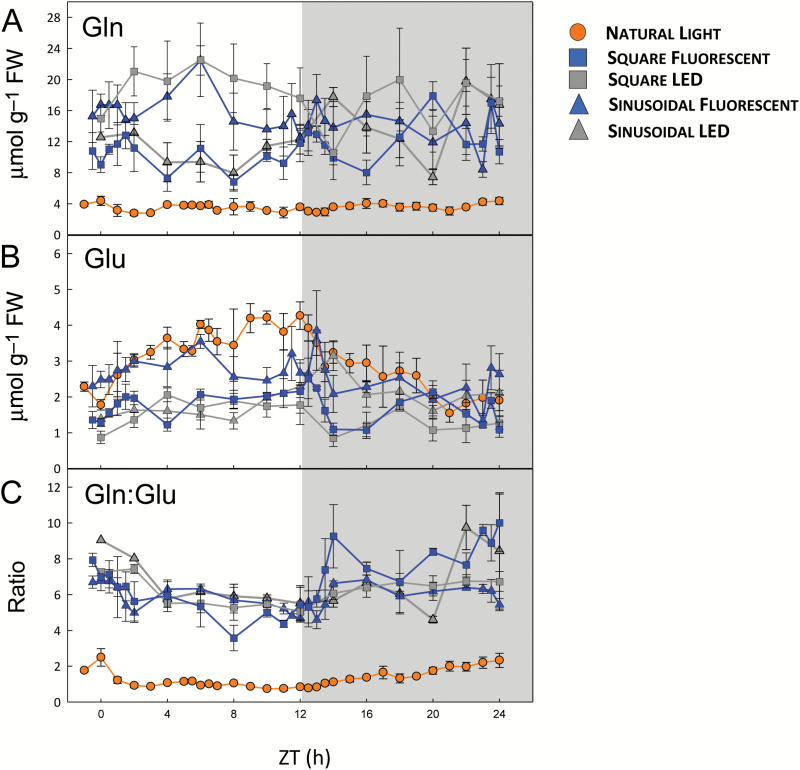
Diurnal profiles of glutamine (Gln) and glutamate (Glu) in arabidopsis plants growing in natural or artificial light with a 12-h photoperiod. *Arabidopsis thaliana* Col-0 plants were grown in a naturally illuminated greenhouse around the vernal equinox in 2012 (orange circles) and in controlled environment chambers with a 12-h photoperiod and daily light integral (DLI) of 7 mol m^−2^ d^−1^. Artificial illumination was provided by white fluorescent tubes (blue symbols) or LEDs (grey symbols), with either a constant (squares) or sinusoidal (triangles) light profile. Rosettes were harvested from 4-week-old plants throughout a 24-h diurnal cycle for metabolite analysis. (A) Gln and (B) Glu were measured by HPLC, and the Gln:Glu ratio is shown in (C). Data are means ±SD (*n*=3 for LED conditions and *n*=4 for the others). ZT, zeitgeber time (hours after dawn).

We compared the diurnal profiles of individual metabolites in the various light regimes to better understand the metabolic differences between plants grown with natural versus artificial lighting. We first inspected amino acids, because the PCA revealed that many amino acids were contributing to the separation. Notable differences were observed for individual amino acids. Thr, Ser, and Met (also ethanolamine) were higher during both day and night in the plants grown under natural light, while Gln was 2- to 5-fold lower (Fig. 2A and Supplementary Fig. S8). Other amino acids showed differences only during the day (Glu, Arg, and Lys) or only at night (Ala and His). The Gln:Glu ratio, which is considered to be a marker for N limitation (Stitt and Krapp, 1999; Foyer *et al.*, 2003; Fritz *et al.*, 2006), was 3- to 4-fold lower in the plants grown under natural light (Fig. 2C) throughout the light–dark cycle. Likewise, shikimate, the precursor of the aromatic amino acids, was up to 2-fold lower (Supplementary Fig. S8).

In addition to reduced nitrogen (NH_4_^+^), amino acid synthesis requires C-skeletons in the form of organic acids (Nunes-Nesi *et al.*, 2010; Foyer and Zhang, 2011). Pyruvate and all of the measured tricarboxylic acid (TCA) cycle intermediates except 2-oxoglutarate (2-OG) were 2 to 6-fold lower in the plants grown under natural light throughout the light–dark cycle, and 2-OG was lower during the night (see Supplementary Fig. S8). In the plants grown under artificial light, malate, fumarate, and 2-OG accumulated during the day and decreased at night, while citrate showed the opposite pattern. Qualitatively similar, but less pronounced, diurnal changes were seen for these organic acids in the plants grown under natural light. The differences in organic and amino acid levels suggest there were lower rates of amino acid synthesis in the plants grown under natural light.

Starch is the major storage reserve in arabidopsis leaves, and the diurnal cycle of starch synthesis and degradation is carefully regulated to ensure survival through the night and optimal growth (Stitt and Zeeman, 2012; Sulpice *et al.*, 2014; Pilkington *et al.*, 2015). There was little or no increase in starch content up to ZT3 in the natural light conditions (see Supplementary Fig. S8). The lag in starch accumulation was slightly shorter in plants grown under fluorescent or LED lights with a sinusoidal light profile, and much shorter in plants grown under artificial light with a square-wave profile. Despite the initial lag, the plants grown under artificial lights with a sinusoidal light profile accumulated up to one-third more starch by the end of the day (ED) than those grown with constant irradiance, and all of the plants under artificial lights accumulated more starch than the plants grown under natural light. The average starch accumulation rates were 5.6–5.8 µmol[Glc] g^−1^ FW h^−1^ in sinusoidal artificial light, which were higher than in square-wave artificial light (3.9–4.7 µmol[Glc] g^−1^ FW h^−1^) or natural light (3.5 µmol[Glc] g^−1^ FW h^−1^).

Starch was degraded at a near-constant rate throughout the night in all DLI 7 conditions (see Supplementary Fig. S8). By the end of the night, all of the plants had degraded most of their starch reserves, retaining a small residue at dawn (5–8 µmol[Glc] g^−1^ FW), as usually observed in arabidopsis leaves, thereby avoiding C starvation before photosynthesis can resume the following day (Stitt and Zeeman, 2012).

Under natural light, daytime sucrose levels broadly tracked the light levels at the time of harvesting, rising after dawn, plateauing through the middle of the day, and falling towards dusk, then staying low and fairly constant through the night (see Supplementary Figs S1 and S8). In the plants grown under artificial light with a sinusoidal light profile, sucrose also tracked the irradiance during the day, rising to a peak around midday (ZT6) before falling back towards dusk. Under square-wave conditions, sucrose rose sharply in the first 2 h after dawn and then stayed more or less constant throughout the day. Total soluble sugar levels (sucrose + glucose + fructose) showed similar patterns (Supplementary Fig. S8). Throughout the light–dark cycle, the plants grown under natural light had less sucrose than the plants grown under artificial lighting. Some of the intermediates of sucrose synthesis, including fructose 6-phosphate (Fru6P), glucose 1-phosphate (Glc1P), and sucrose 6′-phosphate (Suc6P), were also lower in the plants grown under natural light, as was uridine 5′-diphosphoglucose (UDPGlc), but not Glc6P (Supplementary Fig. S8).

### The effect of diurnal light profile on carbon metabolism at DLI 7

**Fig. 3.**
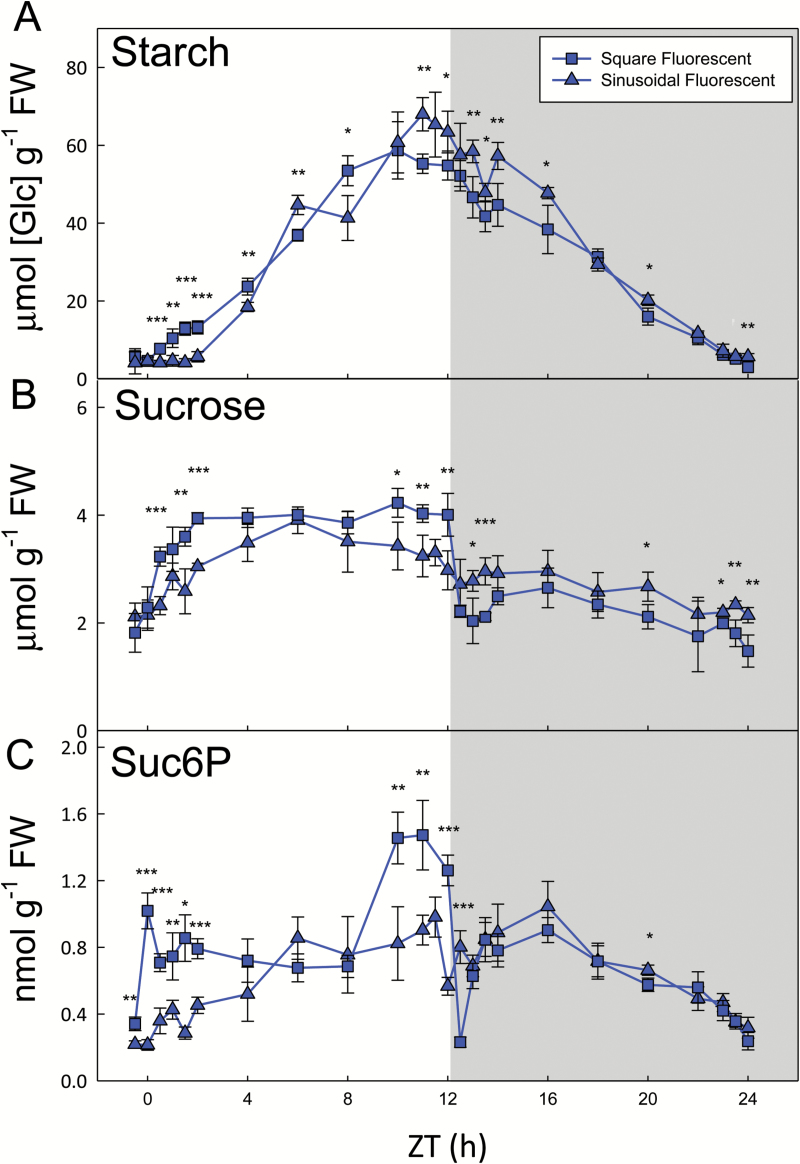
Carbohydrate content of arabidopsis plants grown with constant or sinusoidal fluorescent light profiles. *Arabidopsis thaliana* Col-0 plants were grown in controlled environment chambers with a 12-h photoperiod and daily light integral (DLI) of 7 mol m^−2^ d^−1^. Illumination was provided by white fluorescent tubes with either a constant (squares) or sinusoidal (triangles) light profile, and rosettes were harvested from 4-week-old plants throughout a 24-h diurnal cycle for metabolite analysis. (A) Starch and (B) sucrose were measured enzymatically, and (C) sucrose 6′-phosphate (Suc6P) was measured by LC-MS/MS. Data are means ±SD (*n*=4). At each time point, significant differences between the two conditions are indicated as follows: *P<0.05, **P<0.01, ***P<0.001 (Student’s *t*-test). ZT, zeitgeber time (hours after dawn).

To assess the sensitivity of metabolism to different components of the light regimes (i.e. diurnal light profile and spectral quality), we performed pairwise comparisons between treatments. Under fluorescent lights, the greatest differences in metabolite levels between plants grown under sinusoidal versus square light regimes were often seen around dawn and dusk (see Supplementary Fig. S8). As noted above, there was a pronounced lag (up to ZT2) in net starch synthesis and a plateauing towards dusk in the sinusoidal regime, while starch began to accumulate within 30 min of illumination and continued to increase for most of the day under constant light conditions (Fig. 3A). Sucrose rose after dawn and then tracked irradiance under both conditions, but responded very differently at dusk (Fig. 3B). Sucrose levels were barely affected by dusk in the sinusoidal light regime, but halved within 30 min of the sudden light-to-dark transition under square-wave conditions, before recovering over the next 1–2 h. Suc6P levels also dropped precipitously at dusk in the square-wave regime (Fig. 3C), as did some of the other intermediates of sucrose synthesis (Supplementary Fig. S8). Trehalose 6-phosphate (Tre6P) is thought to be a signal of sucrose status (Lunn *et al.*, 2006; Figueroa and Lunn, 2016). The levels of Tre6P broadly tracked those of sucrose in both the sinusoidal and square-wave light regimes (Supplementary Fig. S9A, B). Tre6P was significantly correlated (*P*<0.001) with sucrose under both conditions, but with a slightly higher sucrose:Tre6P ratio in the square-wave conditions (Supplementary Fig. S9C).

### The effect of light spectral quality on carbon metabolism (DLI 7)

**Fig. 4.**
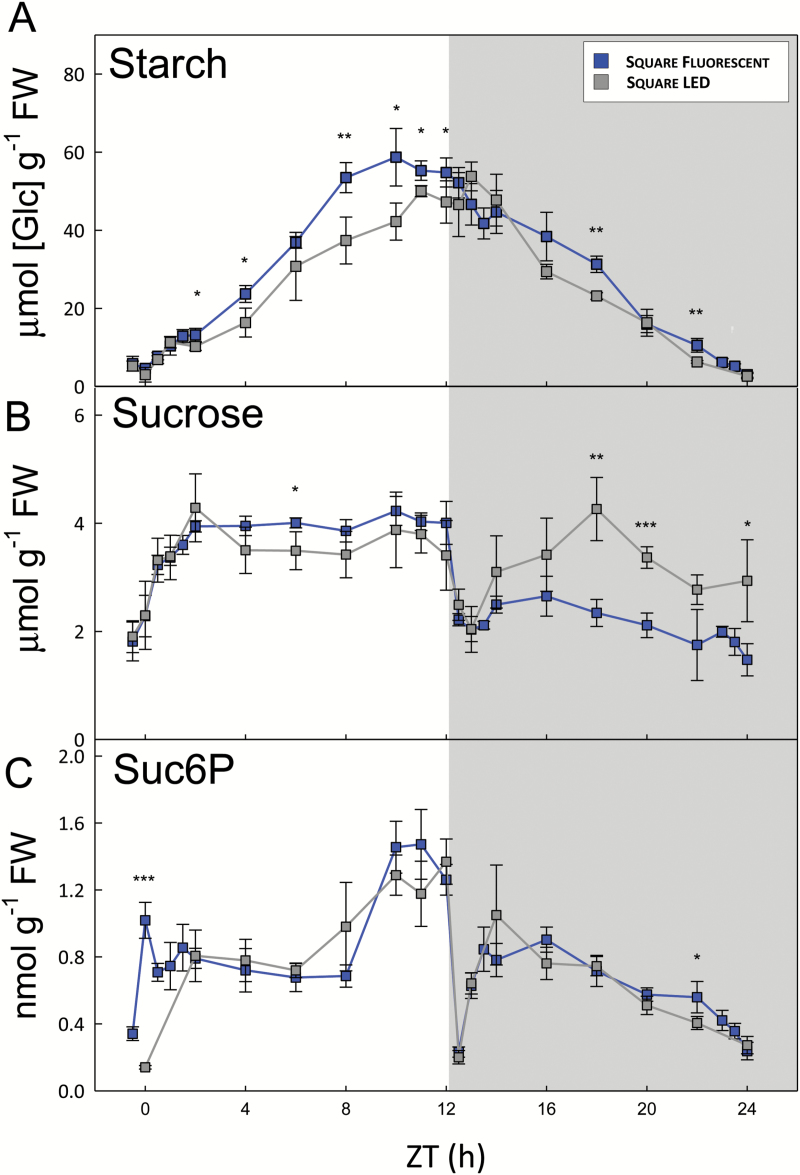
Carbohydrate content of arabidopsis plants grown with constant irradiance under fluorescent or LED lights. *Arabidopsis thaliana* Col-0 plants were grown in controlled environment chambers with a 12-h photoperiod and daily light integral (DLI) of 7 mol m^−2^ d^−1^. Illumination was provided by either white fluorescent tubes (blue) or LEDs (grey) with a constant irradiance during the day, and rosettes were harvested from 4-week-old plants throughout a 24-h diurnal cycle for metabolite analysis. (A) Starch and (B) sucrose were measured enzymatically, and (C) sucrose 6′-phosphate (Suc6P) was measured by LC-MS/MS. Data are means ±SD (*n*=4, fluorescent; *n*=3, LED). At each time point, significant differences between the two conditions are indicated as follows: *P<0.05, **P<0.01, ***P<0.001 (Student’s *t*-test). ZT, zeitgeber time (hours after dawn).

We next compared plants grown under fluorescent versus LED lighting. As the differences were fairly consistent between the square-wave and sinusoidal light regimes (see Supplementary Fig. S8), and most controlled environment experiments use constant irradiance during the day, we focus on the square-wave regimes. Overall, there were few significant differences between plants grown with fluorescent versus LED lighting, and where differences occurred they were mostly small. The diurnal patterns of starch accumulation and degradation were very similar (Fig. 4A), although the net rate of starch synthesis under fluorescent lights (4.76 µmol[Glc] g^−1^ FW h^−1^) was about 20% higher than under LED lights (3.92 µmol[Glc] g^−1^ FW h^−1^). Sucrose levels were essentially the same during the day, but up to twice as high in the plants grown under LEDs during the middle of the night (Fig. 4B). Likewise, Suc6P levels were almost identical under both conditions (Fig. 4C). Although Suc6P appeared to rise more rapidly after illumination with fluorescent lights, this probably reflects a slight difference in the sampling schedules around dawn in the two experiments, and Suc6P was not measured at the ZT0.5–ZT1.5 time points in the LED samples due to insufficient material. There were few significant differences in hexose-monophosphate levels, but fructose 1,6-bisphosphate (Fru1,6BP) and glucose 1,6-bisphospate (Glc1,6BP) were up to 2-fold higher in plants grown under fluorescent light (Supplementary Fig. S8). There were few and only minor differences in TCA cycle intermediates, but total amino acid levels were about twice as high in the plants grown under LEDs (Supplementary Fig. S8), mostly due to the 2-fold higher Gln content of these plants (Fig. 2A).

### Comparison of plants grown under natural and fluorescent light at higher irradiance at DLI 12

There was relatively little cloud cover on the day of harvest in the 2015 natural light experiment, so the plants received almost twice as much light over the course of the day (DLI=12 mol m^−2^ d^−1^) as in the 2012 (DLI 7) experiment. It was not feasible to repeat all of the artificial light regimes with a DLI of 12 mol m^−2^ d^−1^, and we decided to simulate the DLI 12 natural light profile using a sinusoidal fluorescent-light regime (see Supplementary Fig. S2B), which should reveal the effect of differences in light spectral quality. The DLI 12 plants had a larger rosette diameter than the DLI 7 plants, but were at a similar developmental stage (Supplementary Fig. S5).

**Fig. 5.**
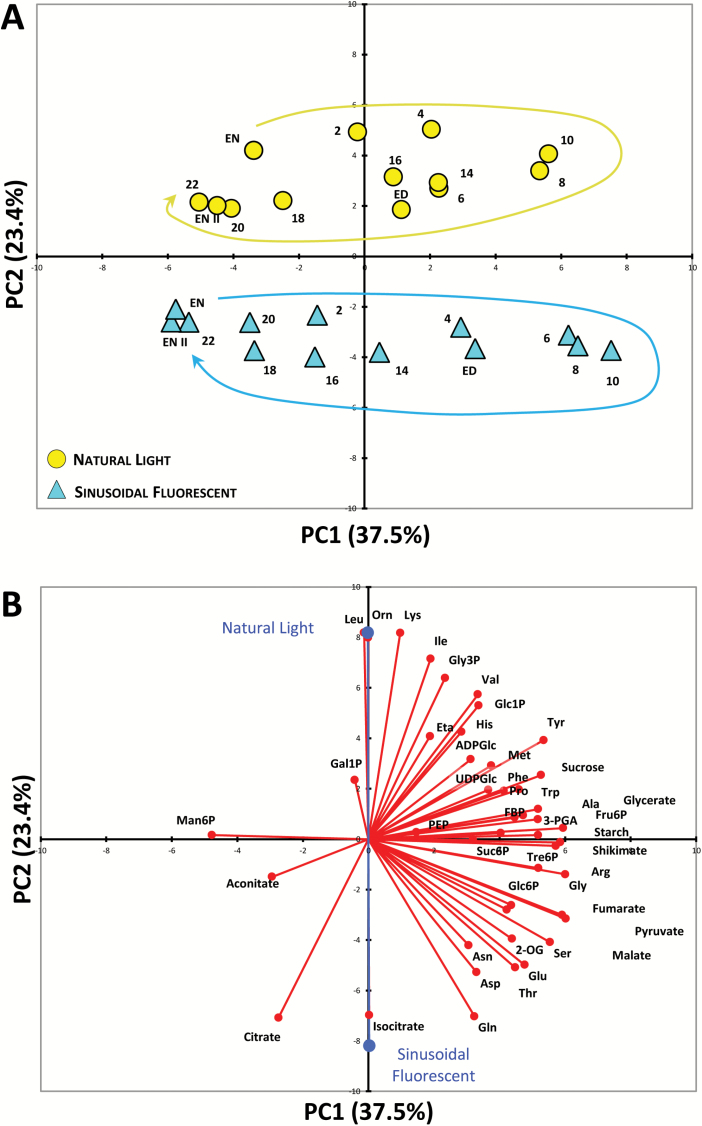
Principal component analysis (PCA) of metabolite data from arabidopsis plants. (A) PCA of metabolite data from plants grown in a naturally illuminated greenhouse (yellow circles) or in a controlled environment chamber with a 12-h photoperiod and daily light integral (DLI) of 12 mol m^−2^ d^−1^. The artificial illumination was provided by white fluorescent tubes with a sinusoidal (blue triangles) light profile during the day. Numbers indicate the time of harvest in hours after dawn (zeitgeber time, ZT); ED, end of day (ZT12); EN I, end of preceding night (ZT0); EN II, end of night (ZT24), and the diurnal trajectories are indicated by the arrows. The percentages of total variance represented by principal component 1 (PC1) and principal component 2 (PC2) are shown in parentheses. (B) The loadings of individual metabolites (red) on the principal components shown in (A) and the (average) loadings of the individual experimental conditions (blue). Glucose and fructose were not included in the PCA due to the very high variability in the data.

PCA showed a clear separation of the plants grown under natural and fluorescent light along the PC2 axis (Fig. 5A), driven mainly by amino acids and TCA cycle intermediates, particularly citrate, isocitrate, and 2-OG (Fig. 5B). The primary separation along the PC1 axis was between light and dark-harvested samples, reflecting differences in starch, sucrose, sugar-phosphates, malate, fumarate, and several amino acids, most notably those associated with photorespiration (Gly, Ser, Glu, and Gln), as well as the aromatic amino acids (Tyr, Trp, and Phe) and their precursor, shikimate. The diurnal trajectories were similar for both plants grown under natural and fluorescent light (Fig. 5A).

The diurnal profiles of starch content were very similar in the plants grown under natural and fluorescent light (see Supplementary Fig. S10). There was a lag of about 2 h after dawn during which there was little or no starch accumulation, after which starch content increased in a linear manner up to about ZT8, and then slowed, reaching a plateau or even starting to decrease in the last 2 h of the day, and then continued on into the first hours of the night. From about 2 h into the night (ZT14), starch was degraded in a linear manner until dawn, when only a small residue (5–7 µmol[Glc] g^−1^ FW) remained. Sucrose broadly tracked irradiance during the day, remained fairly stable during the gradual light–dark transition, and declined gradually during the night, with only minor differences between the two light regimes (Supplementary Fig. S10). The total soluble sugar content (sucrose + glucose + fructose) followed a similar pattern, but was about 10–30% lower in the plants grown under natural light during the first half of the night (Supplementary Fig. S10), reflecting their slightly lower levels of hexose sugars. In the plants grown under natural light, there was a transient spike in the levels of Fru1,6BP, Fru6P, Suc6P, Glc1P, and Glc1,6BP in the middle of the day compared to the plants grown under fluorescent lights (Supplementary Fig. S10). Fru1,6BP, Glc1P, and Glc1,6BP also tended to be higher in the plants grown under natural light during the night.

Plants grown under fluorescent light had about twice as much pyruvate as the plants grown under natural light during the day, and also higher levels of citrate, 2-OG, fumarate, and malate (see Supplementary Fig. S10). The difference in 2-OG was particularly marked, being up to three times higher in the plants grown under fluorescent light and showing a very similar overall pattern to pyruvate. The plants grown under fluorescent lights also had higher total amino acid levels during most of the day (ZT3–ZT12), reflecting their higher levels of all of the major amino acids except Gly (Supplementary Fig. S10). Thr was also higher in the plants grown under fluorescent light, but most of the other minor amino acids had similar profiles in both light regimes, except Lys, Leu, ornithine, and Ile, which were lower in the plants grown under fluorescent light (Supplementary Fig. S10).

### Correlation analysis

**Fig. 6.**
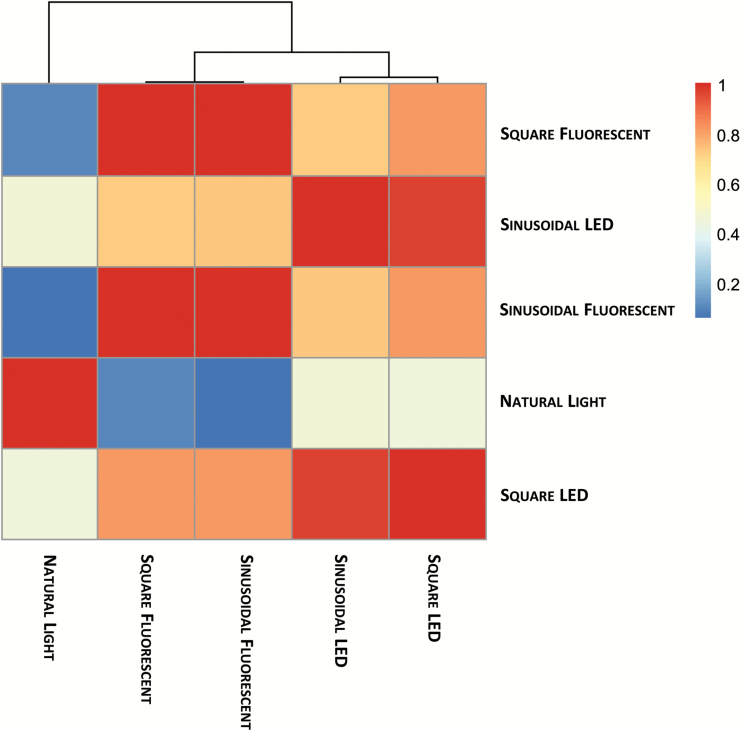
RV coefficient and correlation-based clustering of metabolite time-series data. Metabolite time-series data from *Arabidopsis thaliana* plants grown under natural or artificial light regimes with a 12-h photoperiod and daily light integral (DLI) of 7 mol m^−2^ d^−1^ were analysed by correlation-based clustering. A similarity score was calculated for each pair of growth conditions based on the covariance between the time-series of metabolite data. The results are displayed as a heat map with colours indicating the similarity score, where a value of 0 (blue) indicates no similarity and 1 (red) indicates complete correspondence of the compared covariance structures.

To obtain an overview of the metabolic status of plants grown under the different light regimes, the data from the DLI 7 experiments were subjected to correlation analysis. A similarity score was calculated for each pair of conditions based on the RV coefficient between the covariance matrices calculated from the respective metabolite profiles over the entire light–dark cycle. The similarity score ranges from 0 to 1, with 0 indicating no similarity and 1 indicating complete concordance of the two compared covariance matrices. The pairwise concordance between the covariance of the data profiles can be used to derive a clustering of the investigated light regimes. The fluorescent sinusoidal and square-wave regimes were the most similar to each other (Fig. 6). Likewise, the LED sinusoidal and square-wave regimes were most similar to each other. The fluorescent and LED clusters were also more concordant, and clearly distinct from the natural light dataset. Thus, the light source (i.e. natural versus artificial, and fluorescent versus LED) was the major factor separating the clusters, with the diurnal light profile (sinusoidal versus square) having a relatively minor effect.

**Fig. 7.**
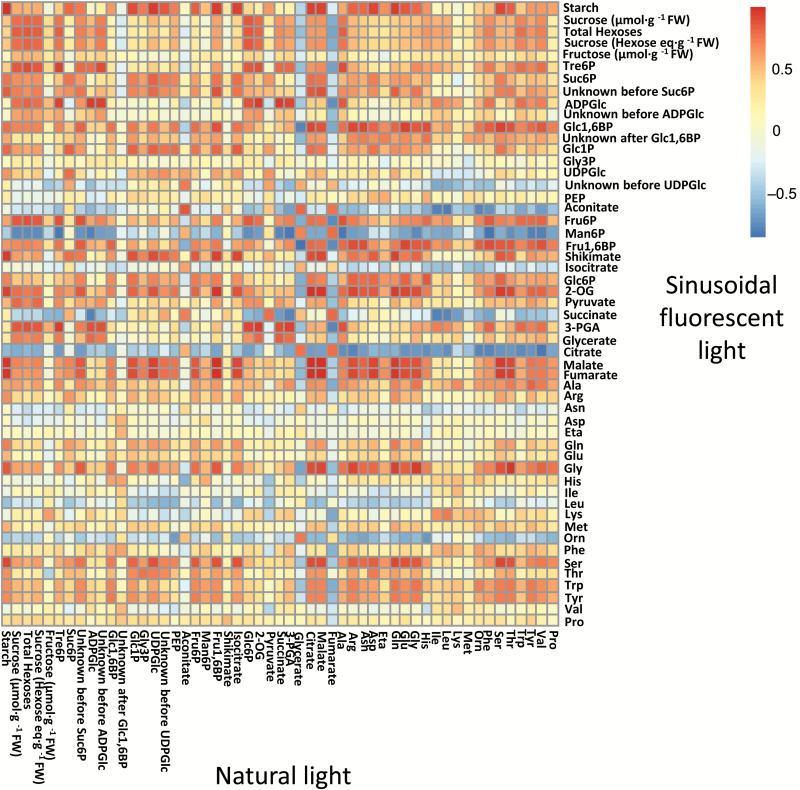
Correlation analysis of metabolite time-series data. Correlation matrix of metabolite time-series data from *Arabidopsis thaliana* plants grown under natural or fluorescent light with a 12-h photoperiod and daily light integral (DLI) of 12 mol m^−2^ d^−1^. The results are presented as a heat map with the correlation score indicated by the shading: red, significant positive correlation after Bonferroni correction; blue, significant negative correlation after Bonferroni correction; white, no significant correlation.

Results from pairwise correlation analysis of the metabolite profiles in the two DLI 12 light regimes are displayed as a heat map in Fig. 7. There were highly significant positive correlations between the plants grown under natural and fluorescent light for many of the intermediates of C metabolism, especially starch, Tre6P, ADPGlc, aconitate, and isocitrate. There was generally a low correlation between individual amino acids in the two light regimes, except for those linked to photorespiration (Ser, Gly, and, to a lesser extent, Gln). Shikimate and 3PGA were negatively correlated between the plants grown under natural and fluorescent light.

## Discussion

### Starch metabolism in different light regimes

Sucrose and starch are the major end products of photosynthesis in most plants. Sucrose is exported from the leaves to support immediate growth, while starch accumulates in the leaf during the day and is then used to maintain the plant through the night (Stitt and Zeeman, 2012). Under C-limiting conditions, the timing and rate of starch breakdown are tightly controlled by the circadian clock (Graf *et al.*, 2010; Scialdone *et al.*, 2013). This allows the plant to predict the length of the approaching night from its recent past history of light–dark cycles, enabling it to make effective use of its starch reserves for growth but not exhaust them before the end of the night. It also allows the plant to adjust the rate of consumption of reserves at night to sudden changes in irradiance, day length, or temperature (Graf *et al.*, 2010; Pyl *et al.*, 2012; Scialdone *et al.*, 2013; Pilkington *et al.*, 2015). The length of the photoperiod is also predictable and has a major influence on the amount of starch accumulated during the day (Britz *et al.*, 1985, 1987; Gibon *et al.*, 2009; Sulpice *et al.*, 2014). However, the amount of light plants receive on any one day is variable and unpredictable in natural environments, as seen in both of our natural light experiments (see Supplementary Figs S1 and S2), and we do not fully understand how plants cope with this variability to control the amount of starch they accumulate during the day.

The maximal levels of starch in the DLI 7 and DLI 12 natural light experiments were 43 and 73 µmol[Glc] g^−1^ FW, respectively (see Supplementary Figs S8 and S10), giving starch:DLI ratios of 6.14 and 6.08. The remarkable similarity of these ratios could arise if, at a given photoperiod, arabidopsis plants partition a fixed percentage of their photoassimilates into starch, irrespective of the rate of CO_2_ fixation, i.e. the ED starch content reflects the total amount of light received during the day. In this respect, arabidopsis, which stores mainly starch in its leaves, may differ from other species that store both sucrose and starch (e.g. spinach and maize) or predominantly sucrose (e.g. wheat). Large changes in photoassimilate partitioning have been observed in these species during the day, which are at least partly linked to changes in the activation state of sucrose-phosphate synthase (Kalt-Torres *et al.*, 1987; Stitt *et al.*, 1987; Lunn and Hatch, 1995; Trevanion *et al.*, 2004).

Our data from arabidopsis plants grown under artificial lights indicate that DLI is not the only factor determining starch content at the ED, as these plants accumulated more starch than those in natural light conditions (see Supplementary Fig. S8), even though the photoperiod (12 h) and DLI (7 mol m^−2^ d^−1^) were the same. These differences may reflect limitations on the speed with which plants can adjust their photoassimilate partitioning in response to fluctuations in irradiance. As a result, partitioning into starch may be sub-optimal during periods when irradiance is fluctuating rapidly and unpredictably. In contrast, in the controlled environment conditions, light was either constant or changing gradually in the same (thus potentially predictable) way every day, perhaps allowing the plants to optimize their partitioning throughout the day and so accumulate more starch. It is interesting to note that in the DLI 12 experiments, the plants grown under natural-light experienced less pronounced fluctuations in irradiance on the day of harvest than in the DLI 7 natural light experiment, and the plants grown under natural-light (DLI 12) accumulated a very similar amount of starch to those grown under sinusoidal (DLI 12) fluorescent lights (Supplementary Fig. S10).

A notable feature of the natural and sinusoidal light regimes was the sigmoidicity of the starch profiles. In both the DLI 7 and DLI 12 experiments, plants grown under these conditions showed a distinct lag (up to 3 h) in starch accumulation at the beginning of the day (see Supplementary Figs S8 and S10). In contrast, net starch synthesis began shortly after dawn in plants grown under constant light. Sucrose levels rose rapidly in the first hours of the day in all conditions, and then generally tracked the irradiance (Supplementary Figs S8 and S10). The rapid rise in leaf sucrose levels indicated that rates of photosynthetic CO_2_ assimilation in the first hour of the day were sufficient to support net carbohydrate accumulation in the leaf, presumably in addition to export of sucrose to the roots. Therefore, it is unlikely that the supply of photoassimilate was limiting starch accumulation. Instead, the initial lag in starch accumulation at the beginning of the day in natural and sinusoidal light regimes might be explained by either a delay in the initiation of starch synthesis, or a continuation of starch breakdown after dawn at a rate that matches starch synthesis.The rate of starch synthesis is thought to be controlled mainly via regulation of ADP-glucose pyrophosphorylase. Upon illumination, this enzyme’s activity will be increased by rising availability of its substrates (Glc1P and ATP), changes in the stromal concentrations of its allosteric regulators (3PGA and Pi), and by thioredoxin-mediated redox modulation (Hendriks *et al.*, 2003; Hädrich *et al.*, 2012; Mugford *et al.*, 2014). ADPGlc pyrophosphorylase (and ADPGlc synthesis) is thus expected to be rapidly activated when there is net CO_2_ assimilation in the light, implying that starch synthesis should also be rapidly initiated, unless the activities of the starch synthesizing enzymes are restricted in some other way. So far, there is little evidence that the starch synthases and branching enzymes are regulated. Thus, it seems more likely that starch synthesis is rapidly initiated after dawn but, under natural and sinusoidal light conditions, synthesis is equally matched by starch degradation for up to 3 h, resulting in the observed lag in starch accumulation. It is not known how starch degradation is regulated in the light. However, we can infer that degradation in the light is inhibited by high light, as there was little or no lag in starch accumulation in plants that were exposed to maximal irradiance immediately after dawn in the square-wave light regimes.

During the middle of the day, the plants grown in sinusoidal artificial light regimes had slightly higher rates of starch accumulation than those in constant light (see Supplementary Fig. S8), most likely due to higher rates of CO_2_ assimilation during periods when the irradiance in the sinusoidal regime was higher than in the constant light conditions (Supplementary Fig. S1). There is also evidence that plants are able to make better use of the prevailing light when this changes in a more gradual manner. In sugar beet leaves, Rubisco was almost fully activated at the maximal irradiance under a sinusoidal light regime, but only 50–60% activated under the same maximal irradiance in a square-wave light regime (Geiger *et al.*, 1991).

In the natural light regimes, starch content started to plateau from about ZT8 (see Supplementary Figs S8 and S10). A similar phenomenon was observed in the square wave DLI 7 and sinusoidal DLI 12 regimes, but in the other DLI 7 light regimes starch accumulation slowed down only in the last 1–2 h of the photoperiod. In square-wave conditions, there was a sharp drop in sucrose and Suc6P levels after the sudden light–dark transition at dusk, followed by a gradual recovery (Figs 3 and 4, Supplementary Figs S8 and S10), as has been observed in previous studies (Martins *et al.*, 2013; Pal *et al.*, 2013). This contrasts with the natural and sinusoidal light regimes, where sucrose levels barely changed around dusk. As starch is the major source of C for sucrose synthesis in the dark, this suggests that starch degradation is rapidly initiated at dusk in natural and sinusoidal light regimes, or may even have already begun in the light. Initiating starch degradation in the final hours of the day might contribute to the decline in net starch accumulation in the natural light conditions. However, in the square-wave DLI 7 regime, where starch content also plateaued, sucrose levels dropped rapidly after dusk, implying that the rate of starch degradation was insufficient to meet demand for C for sucrose synthesis.

Falling light levels and changes in spectral quality as the sun sinks lower in the sky are potential cues for triggering starch degradation before or around dusk, allowing a smooth metabolic transition as the light fades. Plants growing in commonly used square-wave regimes would not receive such cues and so would be less well prepared for the night. The sharp drop in sucrose after dusk suggests that plants growing under these conditions face a nightly C crisis, when their demand for sucrose (for export and maintenance respiration) temporarily exceeds the supply of C, followed by recovery when the rate of starch breakdown becomes sufficient to replenish the pools of sucrose. The transient decrease and recovery of sucrose following abrupt changes from light to dark also indicate that the mechanisms that activate starch synthesis act rather slowly.

### Organic and amino acid metabolism in different light regimes

The separation of plants grown under natural and artificial light in the PCAs of the DLI 7 (Fig. 1) and DLI 12 (Fig. 5) datasets was driven to a large extent by differences in organic and amino acid levels. In the DLI 7 experiments, Ser (also the Ser:Gly ratio) was higher in the plants grown under natural light than in those grown under artificial light, whereas the opposite was observed in the DLI 12 experiments (see Supplementary Figs S8 and S10). This suggests differences in photorespiratory fluxes, or the poise of the mitochondrial glycine decarboxylase reaction, in the plants grown under natural light compared to the corresponding plants grown under artificial lights. However, other observations point to a more general difference in the C/N balance of the plants grown under natural versus artificial light. In particular, the plants grown under natural light (DLI 7) had very low Gln levels and Gln:Glu ratios (Fig. 2), and low levels of the organic acids that provide C-skeletons for amino acid synthesis (pyruvate, 2-OG) or are precursors for C-skeletons (citrate, aconitate, isocitrate, fumarate, malate, and shikimate) (Supplementary Fig. S8). The DLI 12 plants grown under natural light also had low Gln and total amino acid content, along with low citrate, 2-OG, malate, and fumarate (Supplementary Fig. S10). The low levels of organic acids, particularly 2-OG which is considered to be a signal of C/N status (Hodges, 2002), suggested that the supply of C-skeletons was limiting for amino acid synthesis in the plants grown under natural light. However, the plants did not appear to be suffering acute C starvation as levels of Asn were not elevated (Lam *et al.*, 1996); indeed they were, if anything, slightly lower in the plants grown under natural light (Supplementary Figs S8 and S10).

The reason for the apparent C shortage, or more specifically the shortfall in organic acids, in the plants grown under natural light is unclear. Balancing C and N metabolism is likely to be much more challenging for the plants when light levels are varying during the day and from one day to the next, especially as these fluctuations are unpredictable. In the case of the DLI 7 experiments, the two days before the day of harvest were much sunnier (see Supplementary Fig. S1B). It is conceivable that the plants had adapted their C metabolism in response to these successive sunny days, and were then thrown off balance by the cloudy conditions on the day of harvest. The lower Tre6P levels in the plants grown under natural light (Supplementary Figs S8 and S10) could also be relevant. This sucrose signalling metabolite has been implicated in C/N interactions by observations that high Tre6P levels in leaves lead to post-translational activation of phospho*enol*pyruvate carboxylase and nitrate reductase, and increased anaplerotic flux of C into organic acids (Figueroa *et al.*, 2016). Thus, the lower levels of Tre6P in the plants grown under natural light suggest that these plants would have lower phospho*enol*pyruvate carboxylase activity and less flux of C into organic acids.

### LED versus fluorescent lighting

There is considerable interest in replacing fluorescent lighting by LEDs in controlled environment chambers, because they produce a more homogeneous spectral distribution of light and can be tuned to different spectral profiles, including sunlight-like spectra. In addition, their higher efficiency means they have lower energy consumption and running costs, providing economic and environmental benefits. Although a few differences were observed in the metabolite profiles of plants grown under LED versus fluorescent light, these were generally small (Fig. 4, Supplementary Fig. S8). This is quite remarkable given that the LEDs were tuned to give a more sunlight-like spectrum that differed in several ways from the spectrum produced by the fluorescent lights, particularly in the green (495–570 nm) and red (620–750 nm) regions of the spectrum (Supplementary Fig. S4). Green light is able to penetrate deeper into the leaf than red or blue light, so plants exposed to a homogeneous light spectrum can make full use of their photosynthetic capacity in the spongy mesophyll cells, and so have a higher photosynthetic efficiency than plants growing under predominantly red-blue light (Sun *et al.*, 1998; Terashima *et al.*, 2009).

At a global level, the PCA and correlation analyses indicated that the overall metabolic status of the plants grown under LED light was very similar to those grown under fluorescent lights. These results suggest that metabolite data from plants grown under LED lights should be comparable with historical data from experiments that used fluorescent lights.

### Artificial lighting versus natural light regimes

None of the artificial light regimes tested appeared to be a reliable substitute for natural sunlight. Even when we tried to simulate sunlight with LEDs tuned to a give more homogeneous sunlight-like spectrum with a sinusoidal light profile, the metabolite data were more similar to those from the other plants grown under artificial lights than to the plants grown under natural light. Differences in secondary metabolites have also been observed under LED lighting (Darko *et al.*, 2014).

One potentially significant difference between the natural and artificial light sources is the ratio of red:far red (R:FR) light. In direct sunlight, the R:FR ratio varied from 1.31–1.34, depending on the time of day and in agreement with previous measurements (R:FR=1.15; Smith, 1982), whereas fluorescent and LED lights gave much higher R:FR ratios of 3.56–4.82 and 5.22–5.64, respectively (see Supplementary Fig. S4). The higher red-light component of artificial light would be expected to affect phytochrome signalling and phytochrome-dependent processes such as the circadian clock and leaf development. The impact of the R:FR ratio could easily be tested by addition of FR-emitting LEDs to the LED array, and tuning to achieve a lower R:FR ratio that is more similar to sunlight.

In most regions of the globe, sunlight is highly variable within and between days, due to changeable cloud cover. This variability could be another reason for the observed differences between plants grown under natural and artificial light. Short-term fluctuations in light certainly have a negative impact on photosynthetic water-use efficiency, due to the relatively slow responsiveness of stomata, and affect leaf growth rates and anatomy over the longer term (Vialet-Chabrand *et al.*, 2016, 2017). The recent climatic history experienced by a plant can also lead to adaptations that can enhance survival under stress conditions, such as high temperature (Stief *et al.*, 2014). Therefore, it is possible that plants adapt their metabolism to their recent light history in the expectation that similar light conditions will continue in the future. Although this could confer advantages when conditions are stable, there may be a penalty when conditions change from one day to the next, as occurred during our 2012 natural light experiment.

In conclusion, our data suggest that organic acid and amino acid metabolism may be less robust to daily and day-to-day fluctuations in the light regime than starch turnover, which is regulated in a very flexible manner to cope with variable irradiance. Capturing this type of variation in controlled environment experiments is technically challenging but possible (Vialet-Chabrand *et al.*, 2016, 2017), and could be worthwhile for testing the robustness of phenotypes of transgenic plants before committing to expensive field trials.

## Supplementary data

Supplementary data are available at *JXB* online.

Fig. S1. Diurnal irradiance in natural and artificial light regimes at DLI 7.

Fig. S2. Diurnal irradiance in natural and artificial light regimes at DLI 12.

Fig. S3. Light emitting diode (LED) system and spectrum.

Fig. S4. Visible and far-red light spectra of sunlight and artificial (fluorescent and LED) lights.

Fig. S5. Plant morphology on the day of harvest.

Fig. S6. Principal component analysis of metabolite data from plants comparing constant versus sinusoidal light profiles at DLI 7.

Fig. S7. Principal component analysis of metabolite data from plants comparing fluorescent versus LED lighting at DLI 7.

Fig. S8. Diurnal profiles of metabolites in Arabidopsis plants growing in natural or artificial light with a 12-h photoperiod at DLI 7.

Fig. S9. Sucrose and trehalose 6-phosphate content of plants grown with constant or sinusoidal fluorescent light at DLI 7.

Fig. S10. Diurnal profiles of metabolites in Arabidopsis plants growing in natural or fluorescent light with a 12-h photoperiod at DLI 12.

## Supplementary Material

supplementary_figures_S1-S10

## Abbreviations:

2-OG: 2-oxoglutarate
3-PGA: 3-phosphoglycerate
ADPGlc: adenosine 5′-diphosphoglucose
DLI: daily light integral
ED: end of day
EN: end of night
Fru1,6BP: fructose 1,6-bisphosphate
Fru6P: fructose 6-phosphate
Glc1P: glucose 1-phosphate
Glc6P: glucose 6-phosphate
LC-MS/MS: liquid chromatography-tandem mass spectrometry
LED: light emitting diode
Suc6P: sucrose 6′-phosphate
Tre6P: trehalose 6-phosphate
UDPGlc: uridine 5′-diphosphoglucose
ZT: zeitgeber time.
