# Supplementary material for: Getting back to nature: a reality check for experiments in controlled environments
Source: J Exp Bot. 2017 Jun 29;68(16):4463–77. doi: 10.1093/jxb/erx220 (PMC5853417; doi:10.1093/jxb/erx220)
Supplement: supplementary_figures_S1-S10 [file erx220_suppl_supplementary_figures_s1-s10.pdf]

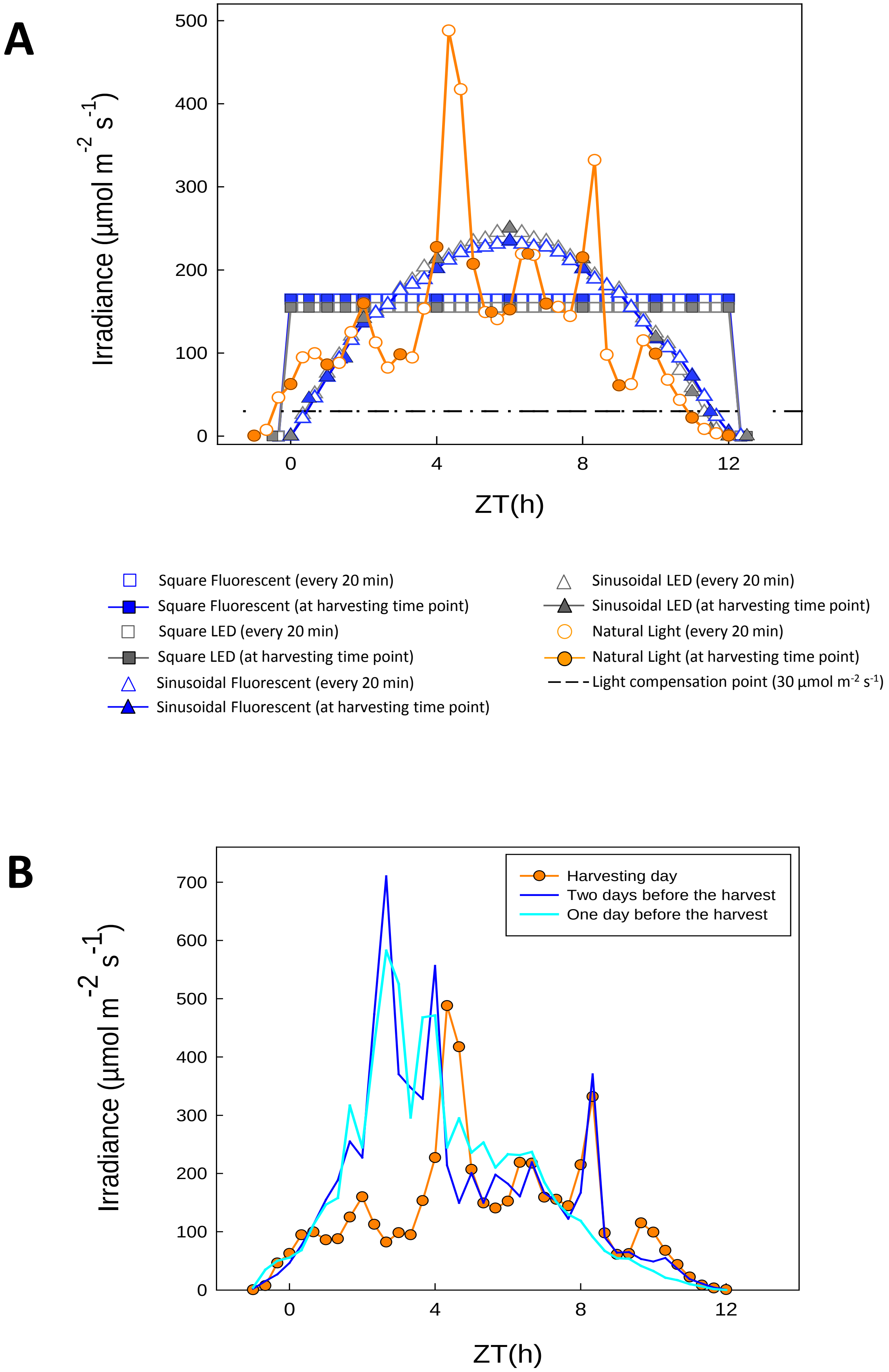
A
Irradiance (μmol m-2 s-1)
500
400
300
200
100
0
0
4
8
12
ZT(h)
Square Fluorescent (every 20 min)
Square Fluorescent (at harvesting time point)
Square LED (every 20 min)
Square LED (at harvesting time point)
Sinusoidal Fluorescent (every 20 min)
Sinusoidal Fluorescent (at harvesting time point)
Sinusoidal LED (every 20 min)
Sinusoidal LED (at harvesting time point)
Natural Light (every 20 min)
Natural Light (at harvesting time point)
Light compensation point (30 μmol m-2 s-1)
B
Irradiance (μmol m-2 s-1)
700
600
500
400
300
200
100
0
0
4
8
12
ZT(h)
Harvesting day
Two days before the harvest
One day before the harvest

Supplemental Figure S1. Diurnal irradiance in natural and artificial light regimes at DLI 7. (A) Irradiance was measured at 20-min intervals (○) in the naturally illuminated greenhouse on the day of harvest at the vernal equinox in 2012, which had a daily light integral (DLI) of 7 mol m^{-2} d^{-1}. Filled circles (●)show the irradiance measured at the time of harvest. Plants were also grown in controlled environment chambers with a 12-h photoperiod and DLI of 7 mol m^{-2} d^{-1}. Illumination was provided by white fluorescent tubes (blue symbols) or LED lights tuned to a sunlight-like spectrum (grey symbols), with either constant (square symbols) or sinusoidal (triangle symbols) light profiles. The dashed line represents the light compensation point for Arabidopsis (30 µmol m^{-2} s^{-1}). (B) shows the measured irradiance in the greenhouse on the day of harvest (●), and on the two preceding days (d-1 —; d-2, —). ZT, zeitgeber time (hours after dawn).

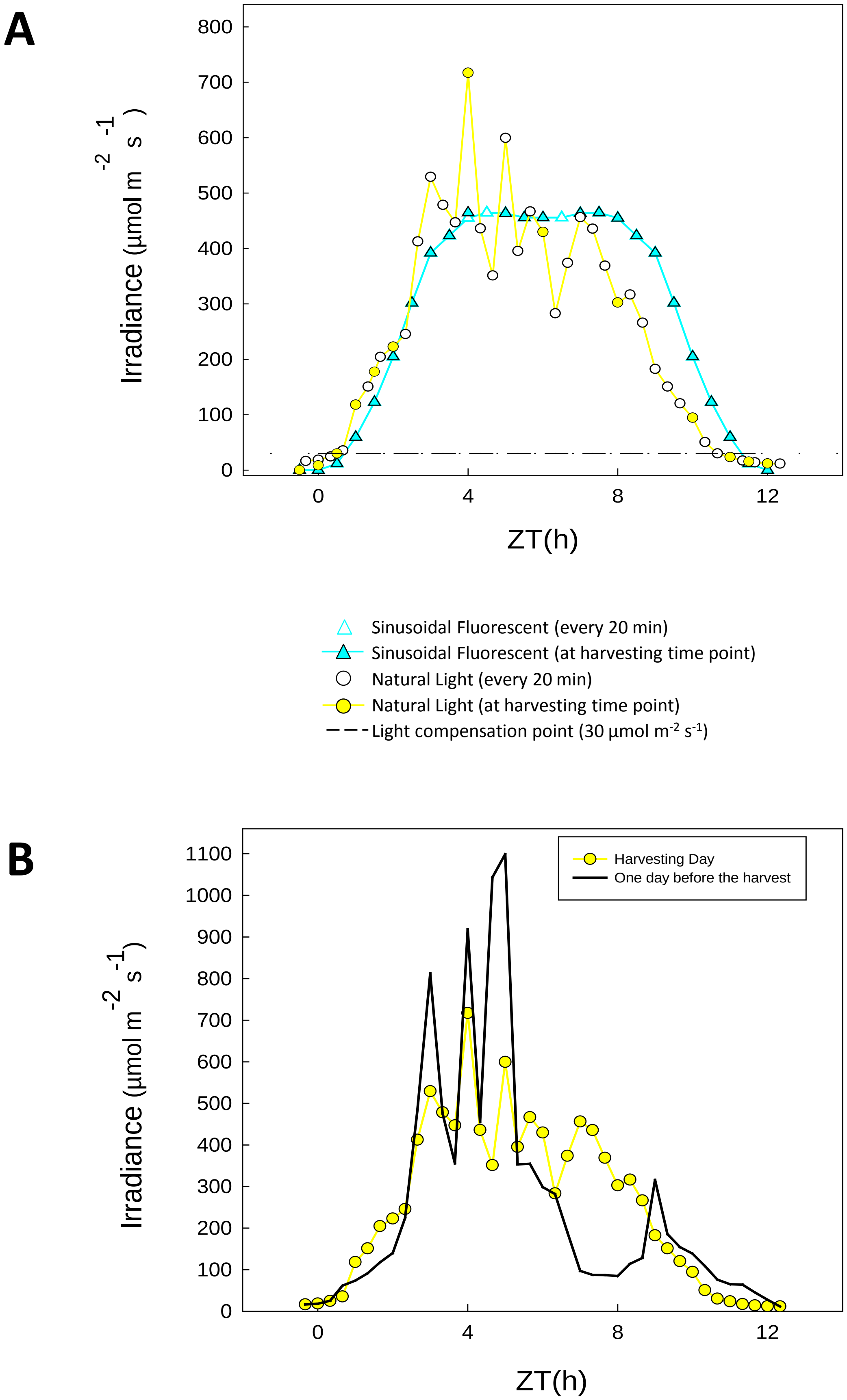
A
800
700
600
500
400
300
200
100
0
Irradiance (µmol m-2 s-1)
0
4
8
12
ZT(h)
Sinusoidal Fluorescent (every 20 min)
Sinusoidal Fluorescent (at harvesting time point)
Natural Light (every 20 min)
Natural Light (at harvesting time point)
Light compensation point (30 µmol m-2 s-1)
B
1100
1000
900
800
700
600
500
400
300
200
100
0
Irradiance (µmol m-2 s-1)
Harvesting Day
One day before the harvest
0
4
8
12
ZT(h)

Supplemental Figure S2. Diurnal irradiance in natural and artificial light regimes at DLI 12. (A) Irradiance was measured at 20-min intervals (○) in the naturally illuminated greenhouse on the day of harvest at the vernal equinox in 2015, which had a daily light integral (DLI) of 12 mol m^{-2} d^{-1}. Filled circles (●) show the irradiance measured at the time of harvest. Plants were also grown in a controlled environment chamber with illumination provided by white fluorescent tubes with a 12-h photoperiod, sinusoidal light profile and a DLI of 12 mol m^{-2} d^{-1} (▲). The dashed line represents the light compensation point for Arabidopsis (30 µmol m^{-2} s^{-1}). (B) shows the measured irradiance in the greenhouse on the day of harvest (●) and on the preceding day (black line). ZT, zeitgeber time (hours after dawn).

A

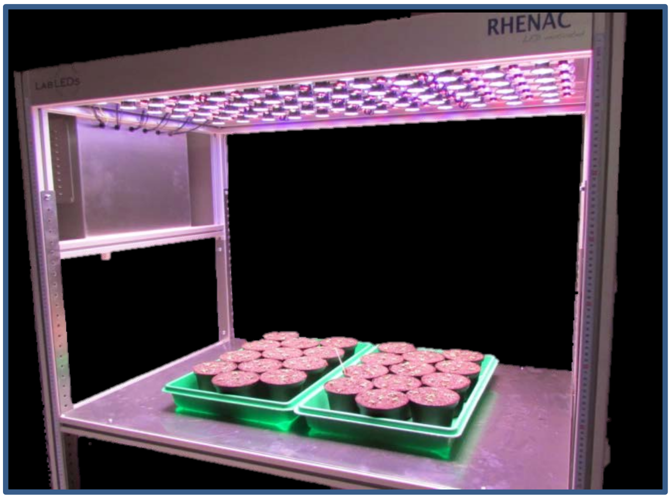

B

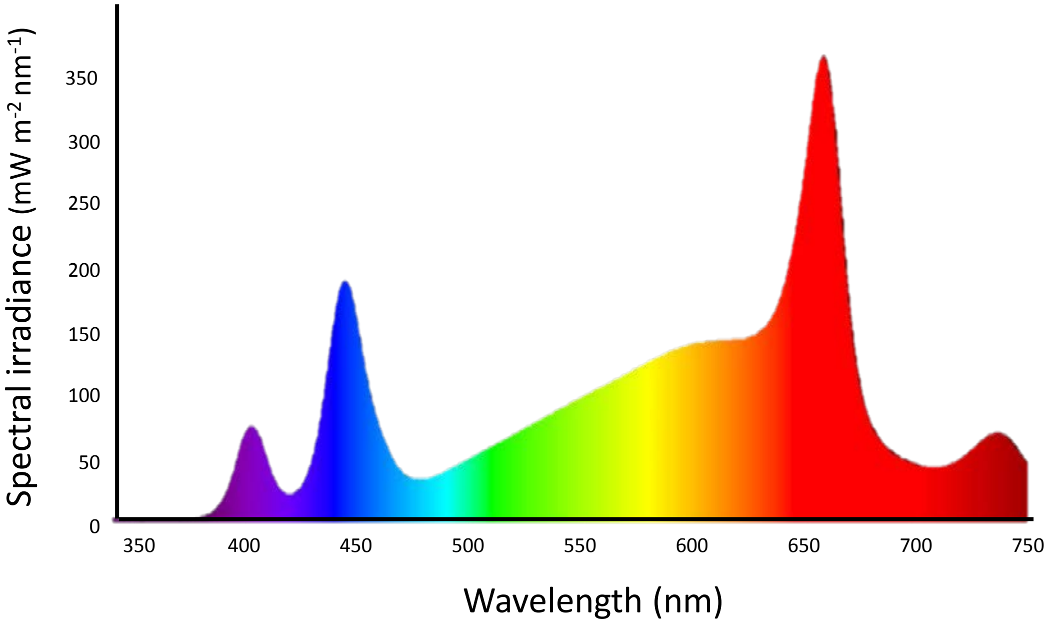

Supplemental Figure S3. Light emitting diode (LED) system and spectrum. (A) mobile R-MU-1002 LED system (RHENAC GreenTec AG, Hennef, Germany; http://www.rhenac-greentec.de/). (B) visible spectrum of LED lights based on standard illuminant D65 (International Commission on Illumination) simulating sunlight in northern/western Europe.

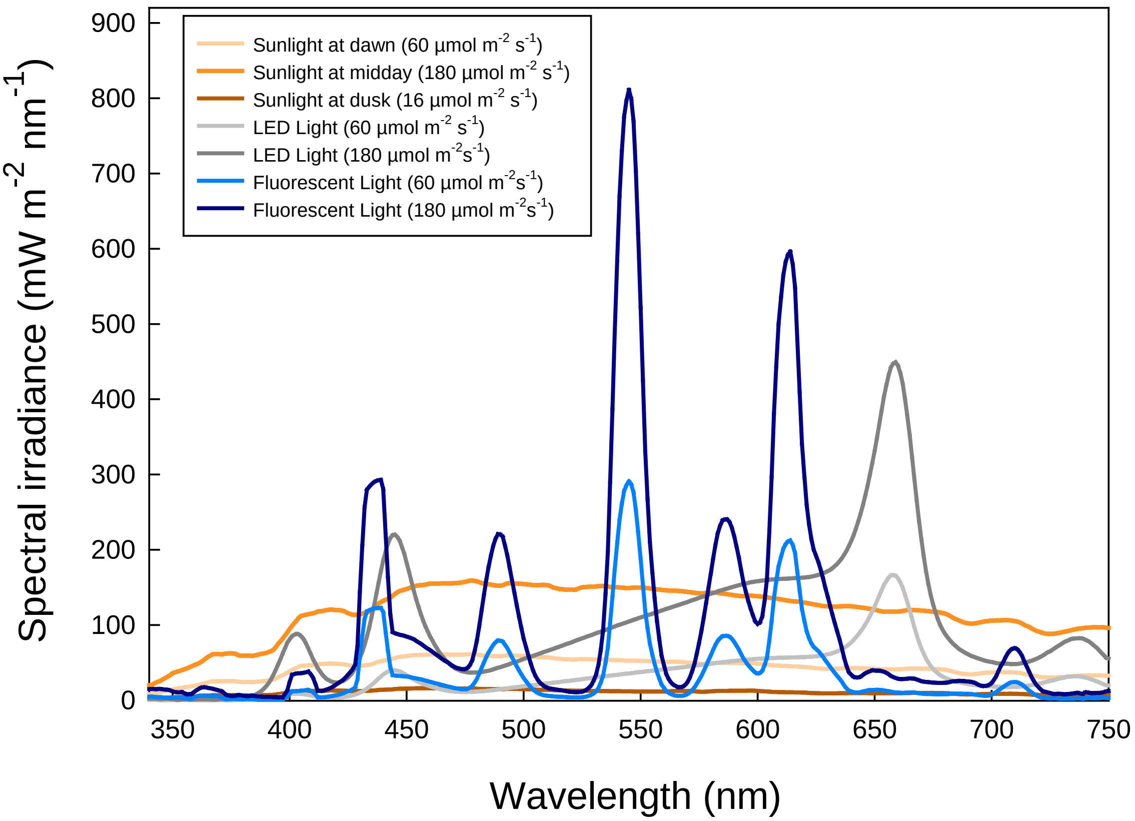

**Light Source**	**Nominal Light Level** (µmol m^{-2} s^{-1})	**R:FR**
Sun (dawn)	60	1.34
Sun (midday)	180	1.31
Sun (dusk)	16	1.34
Fluorescent Light	60	4.82
Fluorescent Light	180	3.56
LED	60	5.22
LED	180	5.64

Supplemental Figure S4. Visible and far-red light spectra of sunlight and artificial (fluorescent and LED) lights. Visible and far-red light spectra are shown for: (i) sunlight at dawn (irradiance 60 µmol m^{-2} s^{-1} , pale orange), midday (180 µmol m^{-2} s^{-1}, orange) and dusk (16 µmol m^{-2} s^{-1}, brown); (ii) white fluorescent tubes at 60 µmol m^{-2} s^{-1} (light blue) and 180 µmol m^{-2} s^{-1} (dark blue); and (iii) LED lights tuned to simulate sunlight at 60 µmol m^{-2} s^{-1} (light grey) and 180 µmol m^{-2} s^{-1} (dark grey). The table shows the ratio of red:far red (R:FR) light for each light source.

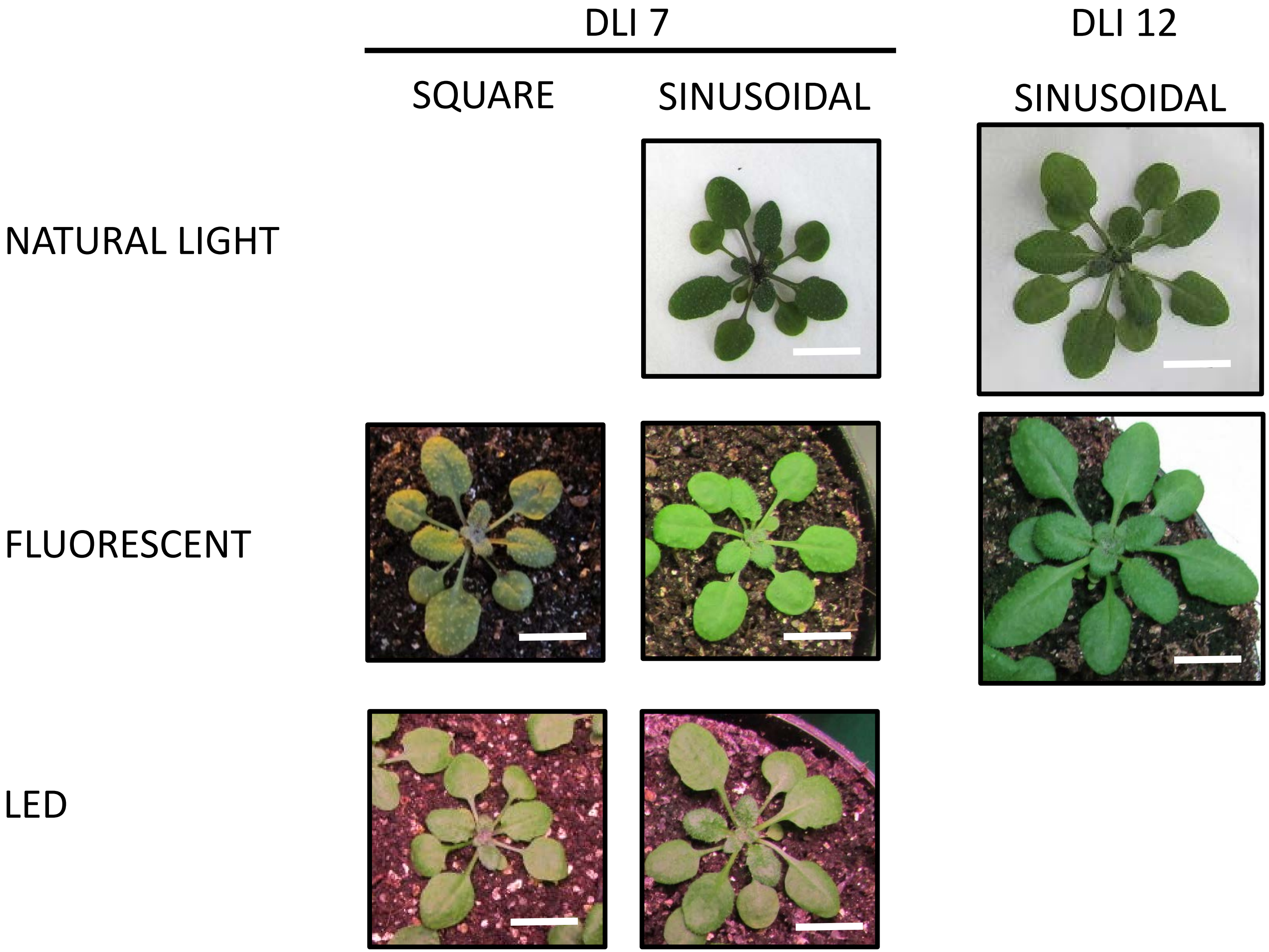

Supplemental Fig. S5. Plant morphology on the day of harvest.
Arabidopsis Col-0 plants were grown in a naturally illuminated greenhouse around the vernal equinox (March 2012 and March 2015), and under five artificial light regimes with a 12-h photoperiod. Artificial illumination was provided by white fluorescent or LED lamps with either square–wave or sinusoidal profile. Plants were harvested at 4 weeks old for metabolite analysis. DLI7, daily light integral = 7 mol m^{-2} d^{-1}; DLI12, daily light integral = 12 mol m^{-2} d^{-1}.

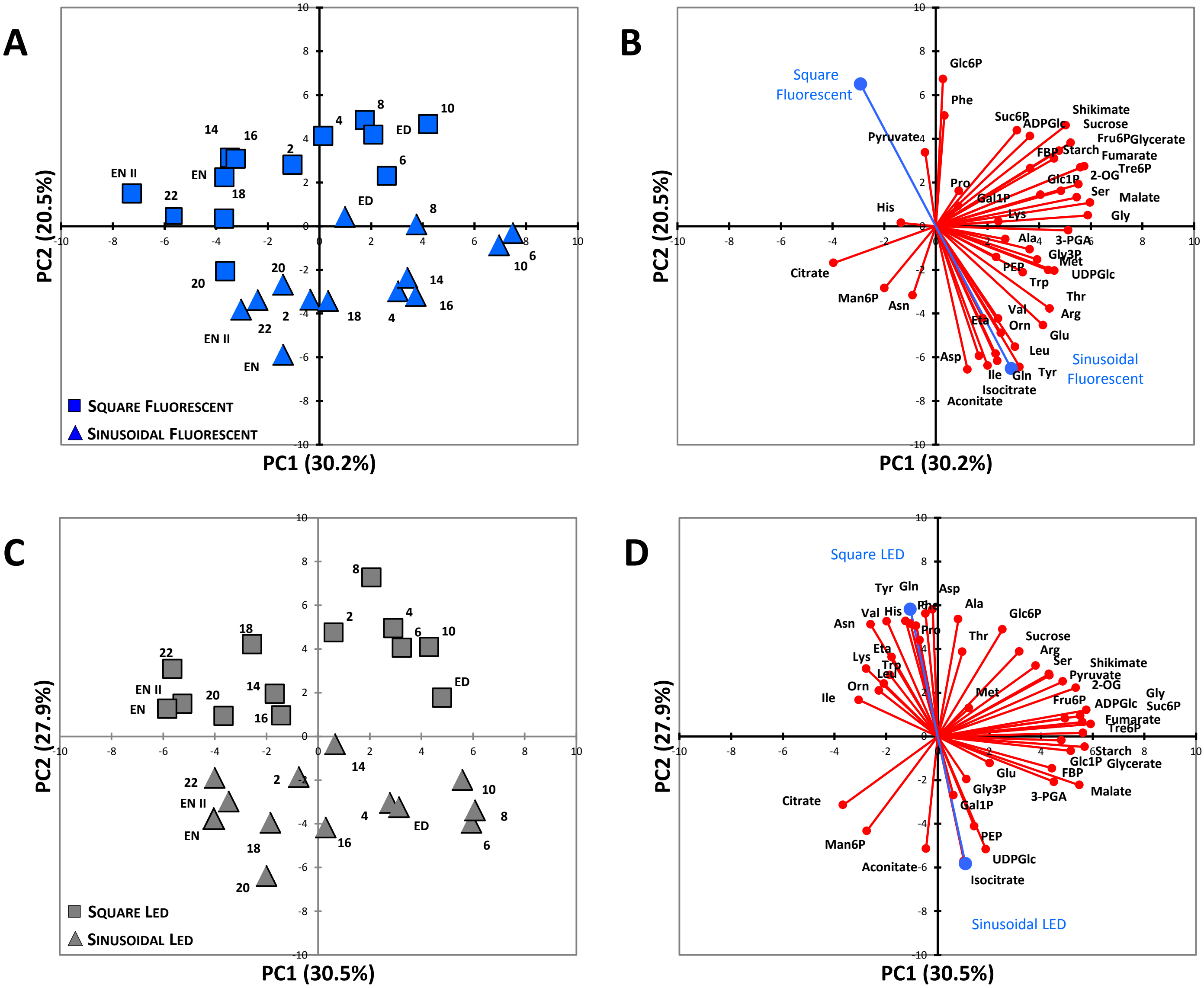

Supplemental Figure S6. Principal component analysis (PCA) of metabolite data from Arabidopsis plants comparing constant *v.* sinusoidal light profiles at DLI 7. PCA of metabolite data from plants grown in controlled environment chambers with a 12-h photoperiod and daily light integral (DLI) of 7 $mol \cdot m^{-2} \cdot d^{-1}$. The artificial illumination was provided by (A,B) white fluorescent tubes (blue symbols) or (C,D) LED lights (grey symbols), with either a constant (square symbols) or sinusoidal (triangle symbols) light profile during the day. Numbers indicate the time of harvest in hours after dawn (zeitgeber time, ZT); ED, end of day (ZT12); EN I end of preceding night (ZT0); EN II, end of night (ZT24). The percentages of total variance represented by principal component 1 (PC1) and principal component 2 (PC2) are shown in parentheses. (B) and (D) show the corresponding loadings of individual metabolites (red) on the principal components shown in (A) and the (average) loadings of the individual experimental conditions (blue).

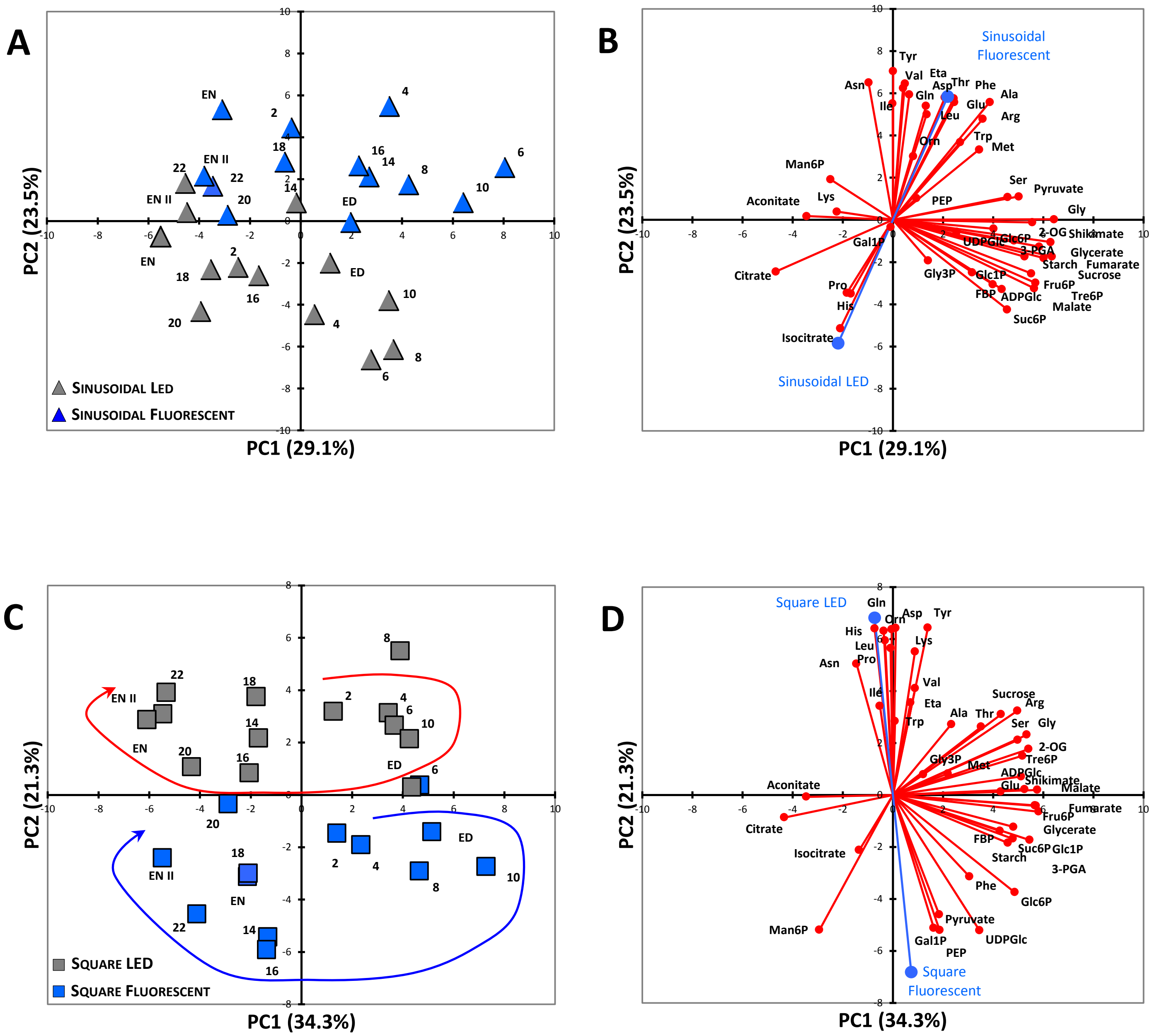

Supplemental Figure S7. Principal component analysis (PCA) of metabolite data from Arabidopsis plants comparing fluorescent *v.* LED lighting at DLI 7. PCA of metabolite data from plants grown in controlled environment chambers with a 12-h photoperiod and daily light integral (DLI) of 7 mol·m^{-2}·d^{-1}. The artificial illumination was provided by white fluorescent tubes (blue symbols) or LED lights (grey symbols), with either (A,B) a sinusoidal (triangle symbols) or (C,D) constant (square symbols) light profile during the day. Numbers indicate the time of harvest in hours after dawn (zeitgeber time, ZT); ED, end of day (ZT12); EN I end of preceding night (ZT0); EN II, end of night (ZT24); the diurnal trajectories are indicated by the arrows. The percentages of total variance represented by principal component 1 (PC1) and principal component 2 (PC2) are shown in parentheses. (B) and (D) show the corresponding loadings of individual metabolites (red) on the principal components shown in (A) and the (average) loadings of the individual experimental conditions (blue).

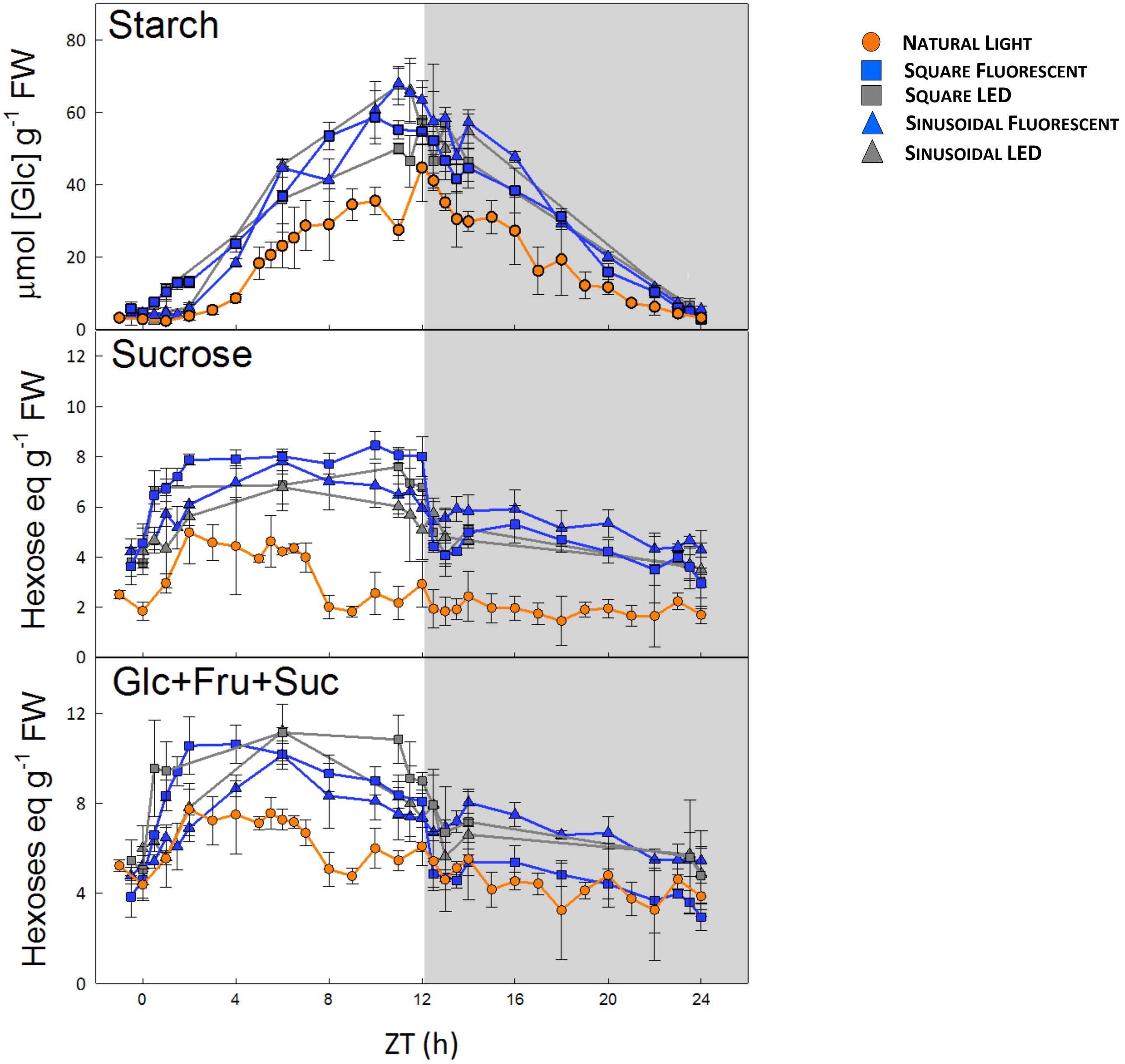

Supplemental Figure S8. (a) Starch and sugars

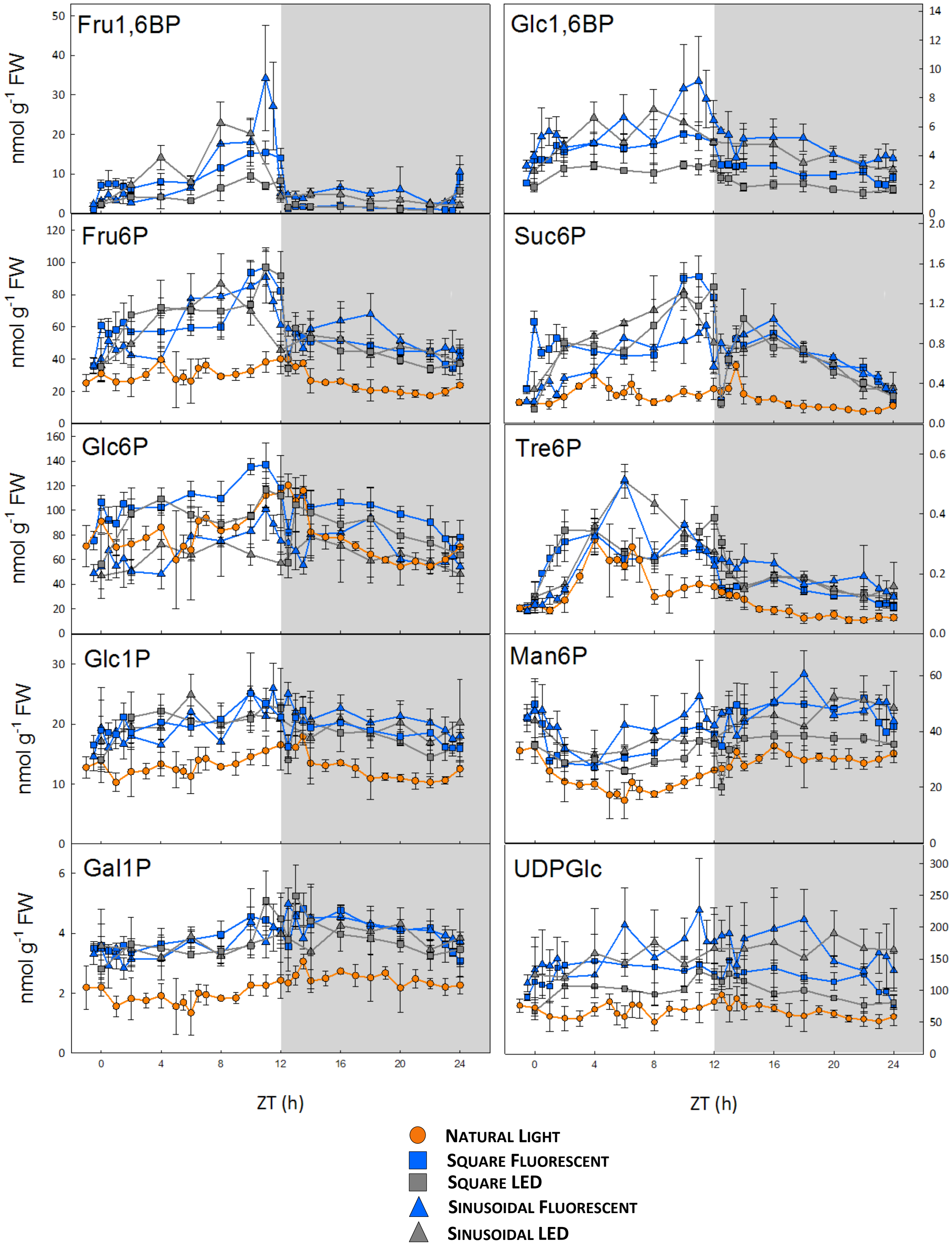

Supplemental Figure S8. (b) Sugar phosphates and nucleotide sugars.

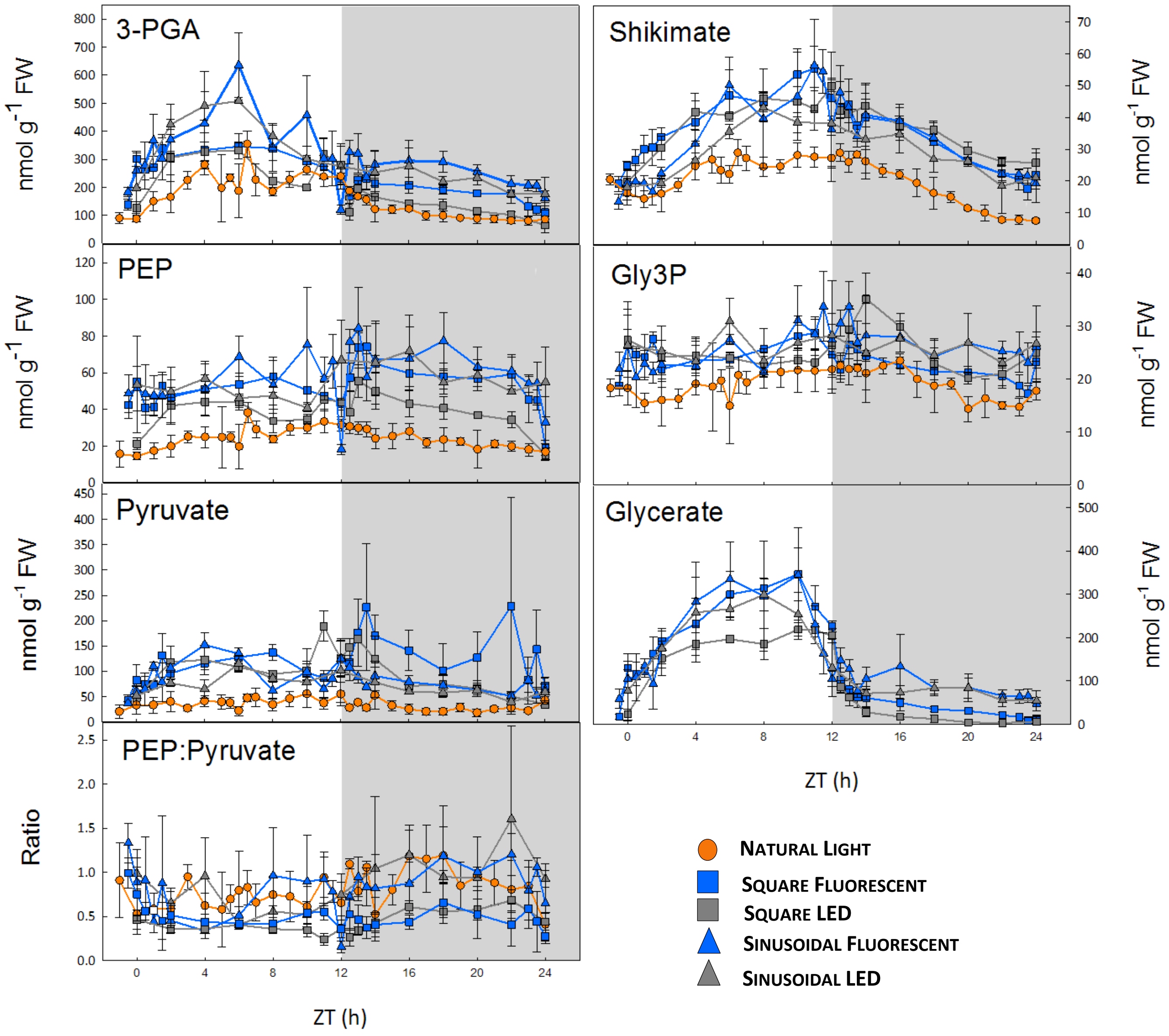

Supplemental Figure S8. (c) Glycolytic intermediates and related metabolites.

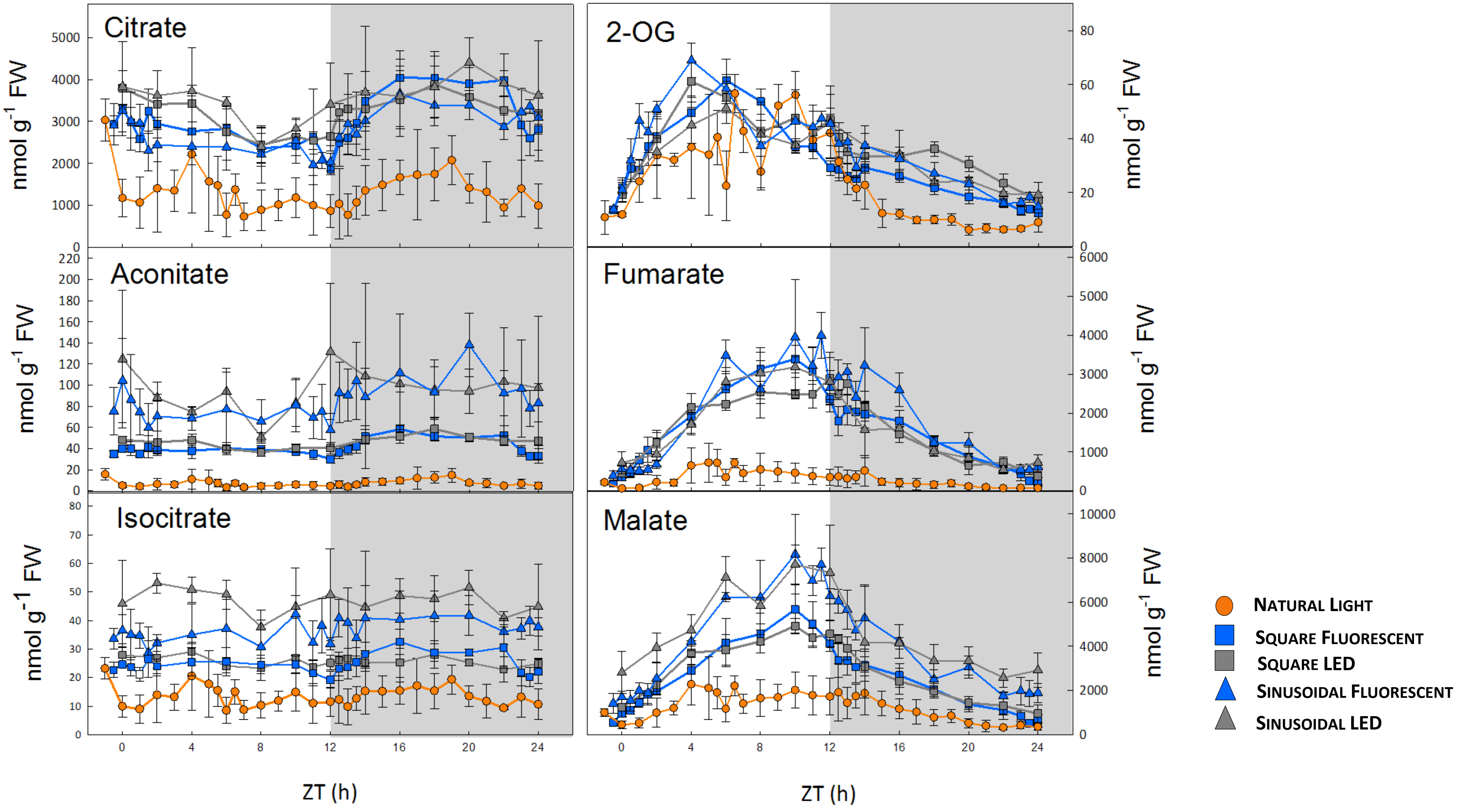

Supplemental Figure S8. (d) TCA cycle intermediates.

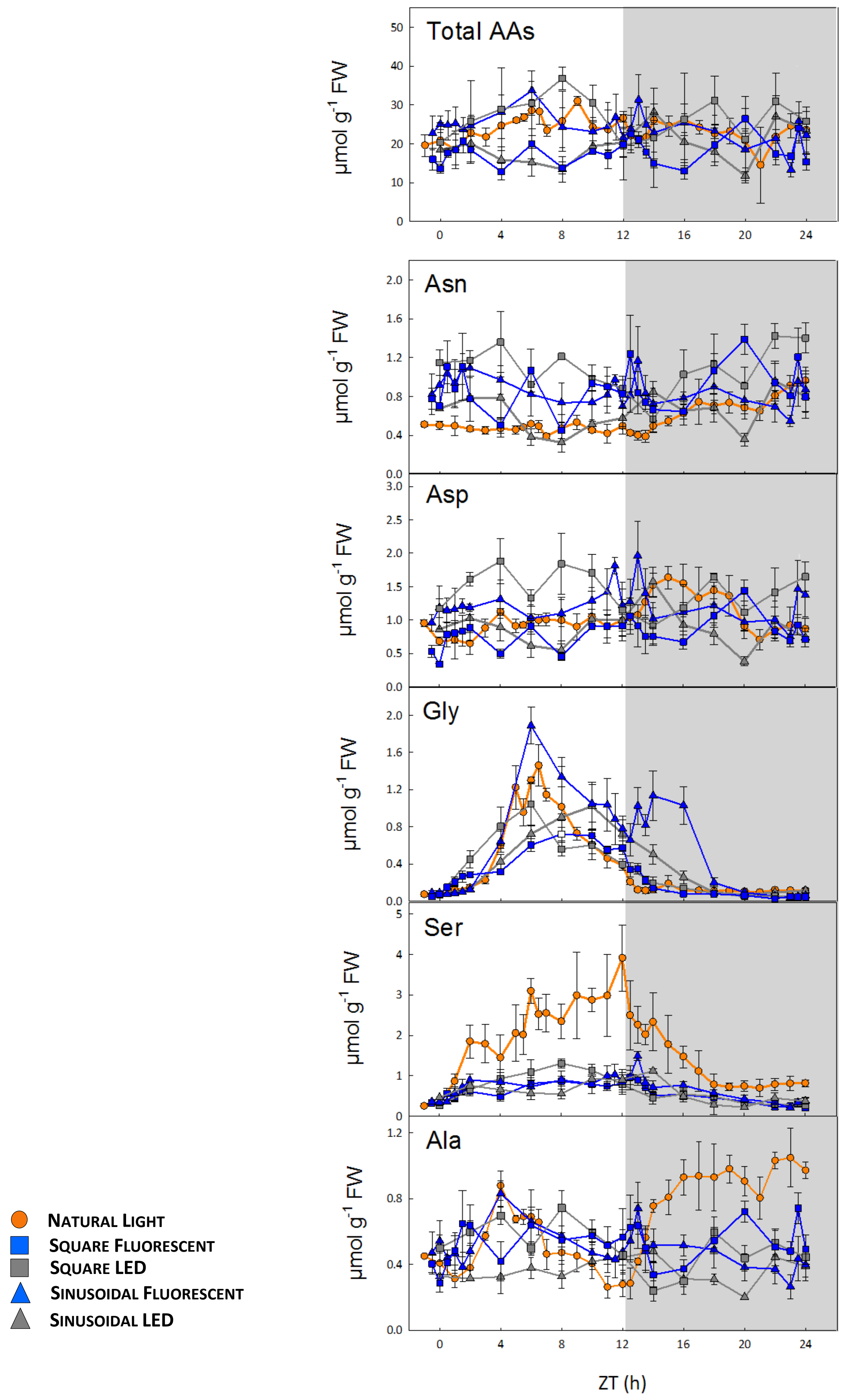

Supplemental Figure S8. (e) Total and major amino acids.

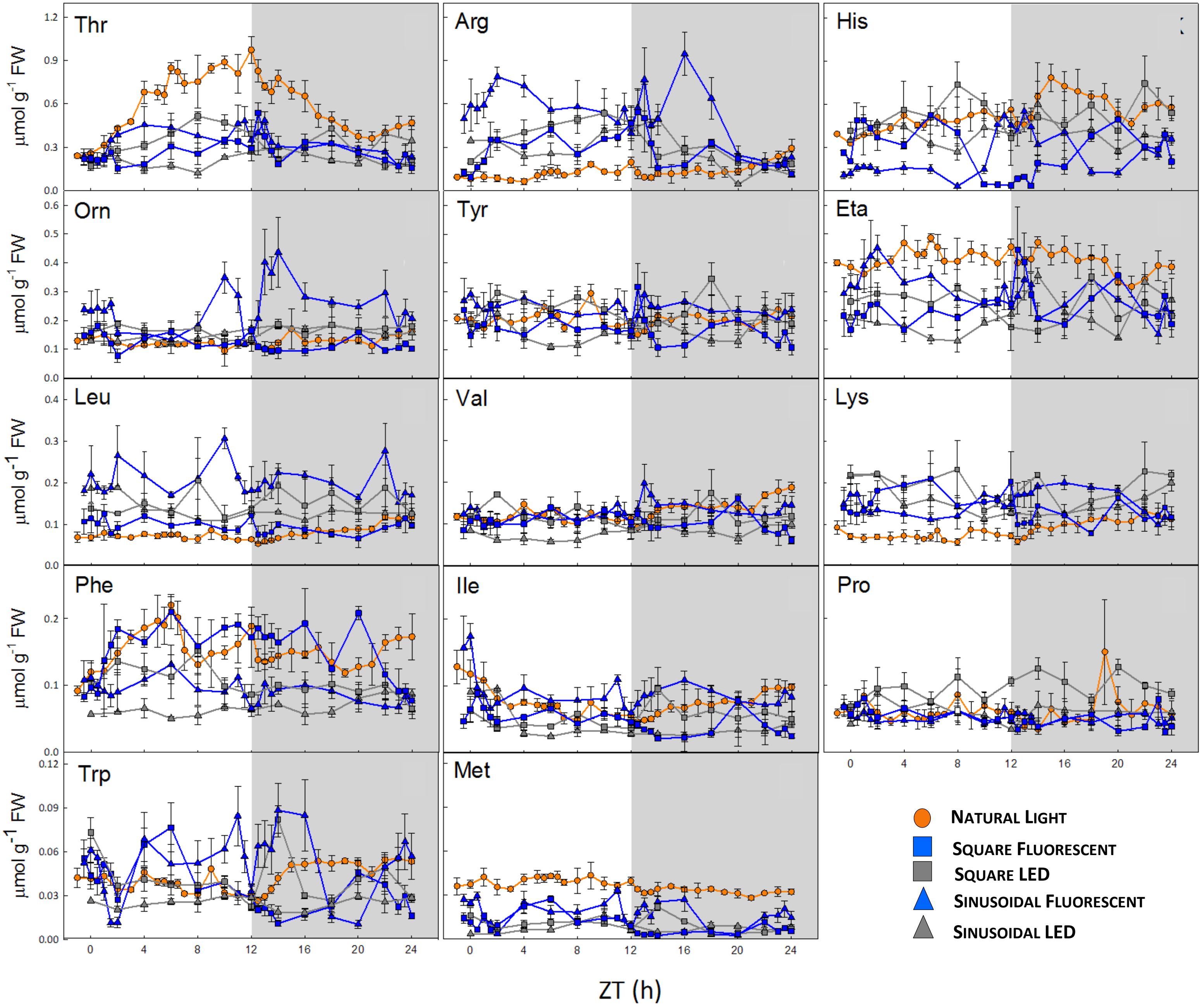

Supplemental Figure S8. (f) Minor amino acids. Eta, ethanolamine; Orn, ornithine

Supplemental Figure S8. Diurnal profiles of metabolites in Arabidopsis plants growing in natural or artificial light with a 12-h photoperiod at DLI 7. *Arabidopsis thaliana* Col-0 plants were grown in a naturally illuminated greenhouse around the vernal equinox in 2012 (●) and in controlled environment chambers with a 12-h photoperiod and DLI of 7 $mol \cdot m^{-2} \cdot d^{-1}$. Artificial illumination was provided by white fluorescent tubes (blue symbols) or LEDs (grey symbols), with either a constant (square symbols) or sinusoidal (triangle symbols) light profile. Rosettes were harvested from 4-week-old plants throughout a 24-h diurnal cycle for analysis of: (a) starch and sugars; (b) sugar phosphates and nucleotide sugars; (c) glycolytic intermediates and related metabolites. (d) TCA cycle intermediates; (e) total and major amino acids; and (f) minor amino acids. Data are mean ± SD (*n*=3 for LED conditions and *n*=4 for the others). ZT, zeitgeber time (hours after dawn).

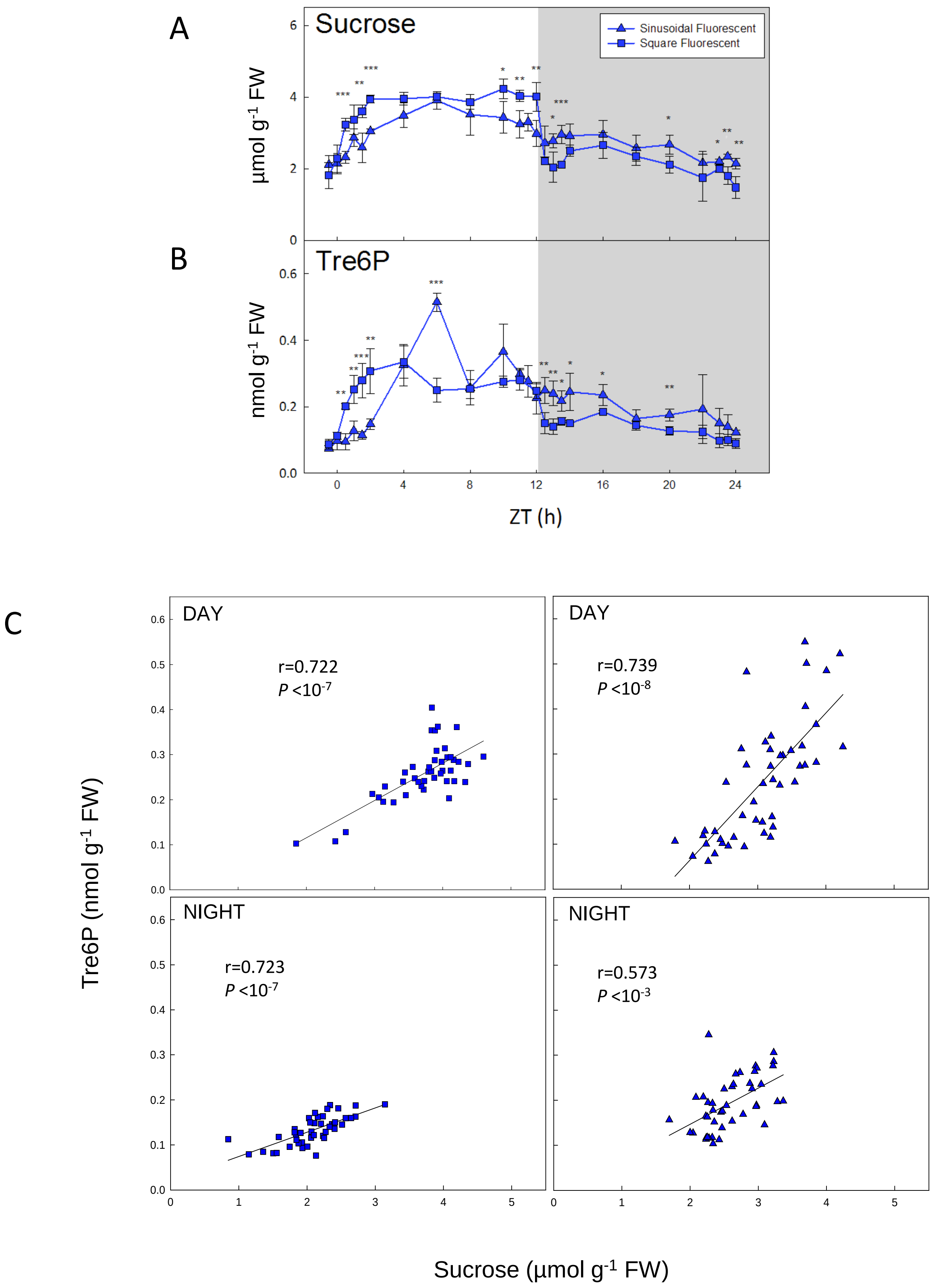
A
Sucrose
Sinusoidal Fluorescent
Square Fluorescent
µmol g-1 FW
B
Tre6P
nmol g-1 FW
ZT (h)
C
DAY
r=0.722
P <10-7
DAY
r=0.739
P <10-8
NIGHT
r=0.723
P <10-7
NIGHT
r=0.573
P <10-3
Tre6P (nmol g-1 FW)
Sucrose (µmol g-1 FW)

Supplemental Figure S9. Sucrose and trehalose 6-phosphate (Tre6P) content of Arabidopsis plants grown with constant or sinusoidal fluorescent light (DLI 7). *Arabidopsis thaliana* Col-0 plants were grown in controlled environment chambers with a 12-h photoperiod and daily light integral (DLI) of 7 $mol{\cdot}m^{-2}{\cdot}d^{-1}$. Illumination was provided by white fluorescent tubes with either a constant (■) or sinusoidal (▲) light regime, and rosettes were harvested from 4-week-old plants throughout a 24-h diurnal cycle for metabolite analysis. (A) sucrose was measured enzymatically and (B) Tre6P was measured by LC-MS/MS. Data are mean ± SD (n=4). At each time point, significant differences between the two conditions are indicated by asterisks: *P<0.05, **P<0.01, ***P<0.001 (Student's t-test). ZT, zeitgeber time (hours after dawn). (C) Scatter plot of Tre6P *v.* sucrose. The Pearson correlation coefficient (r) and the p-value (P) are shown.

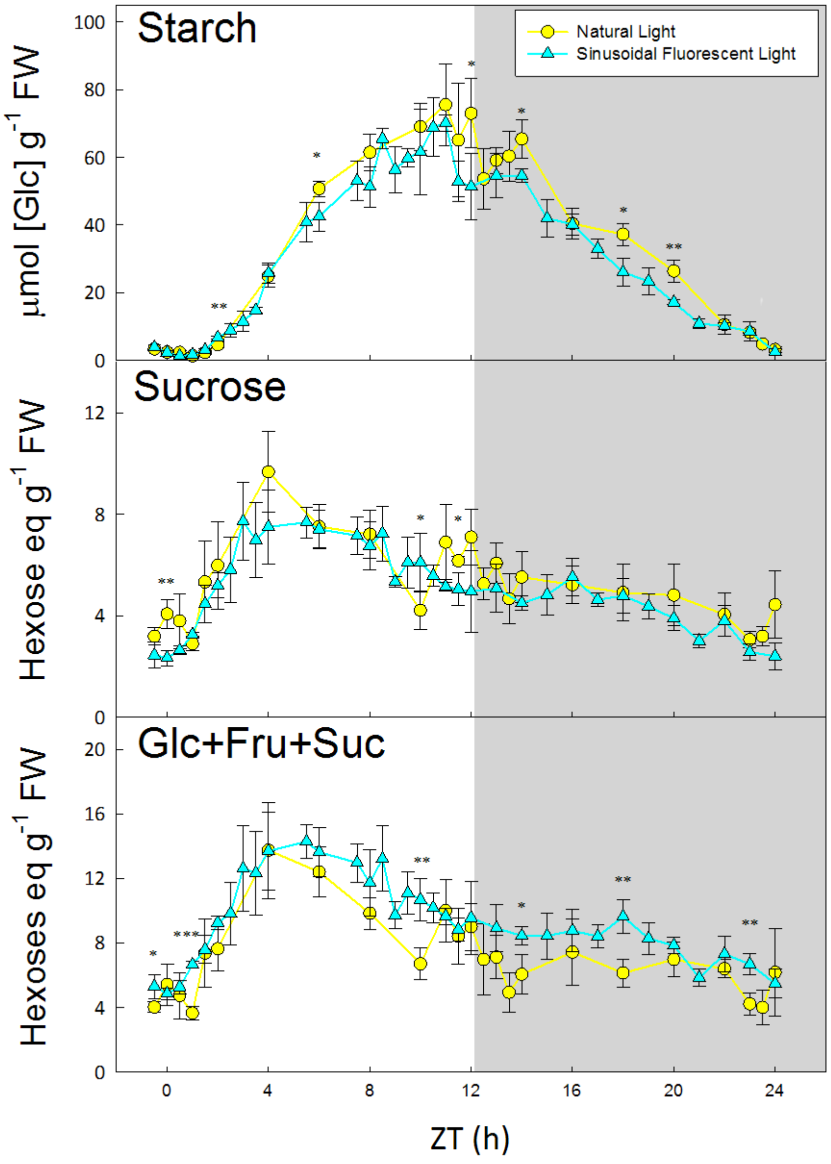

Supplemental Figure S10. (a) Starch and sugars.

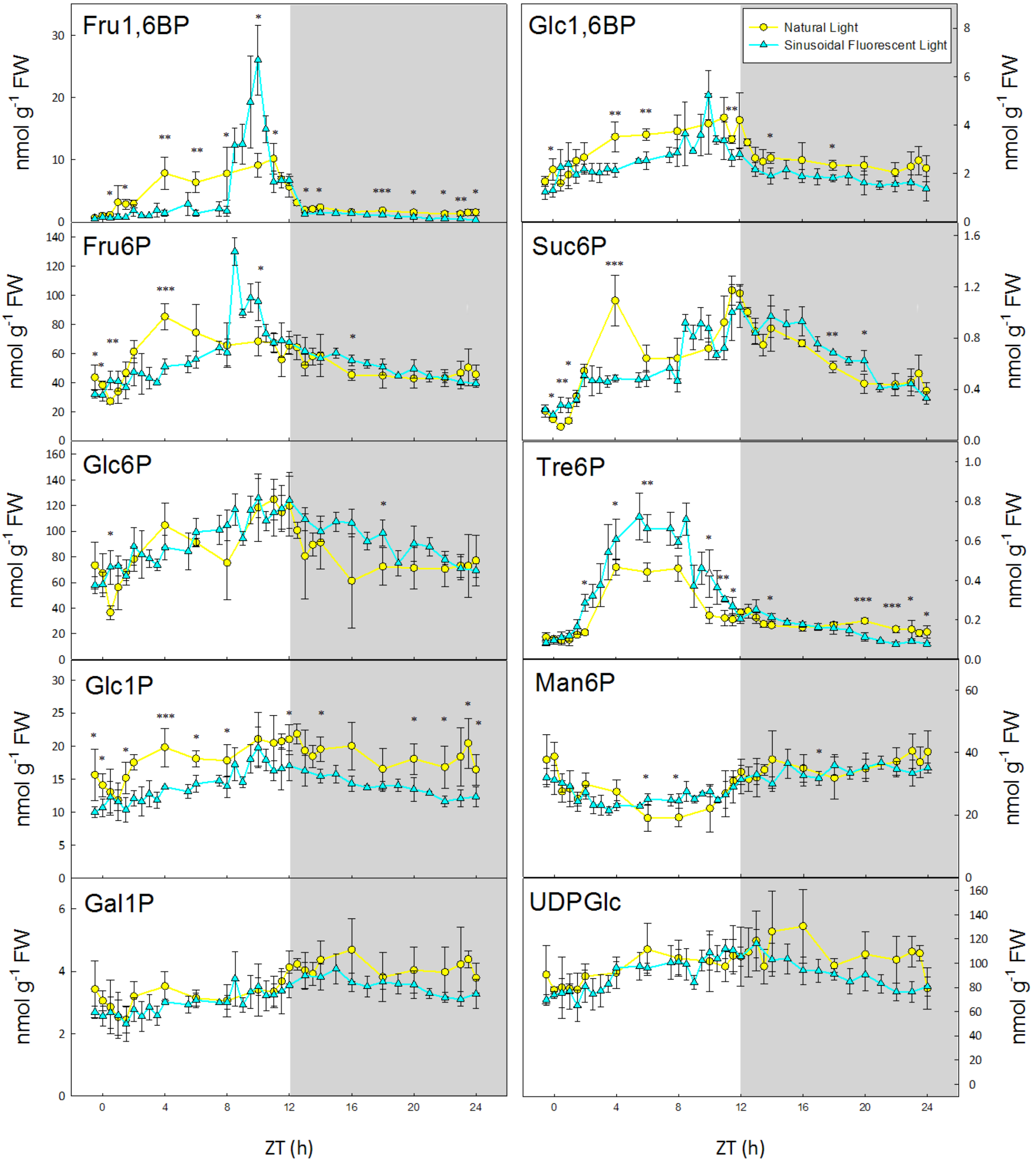

Supplemental Figure S10. (b) Sugar phosphates and nucleotide sugars.

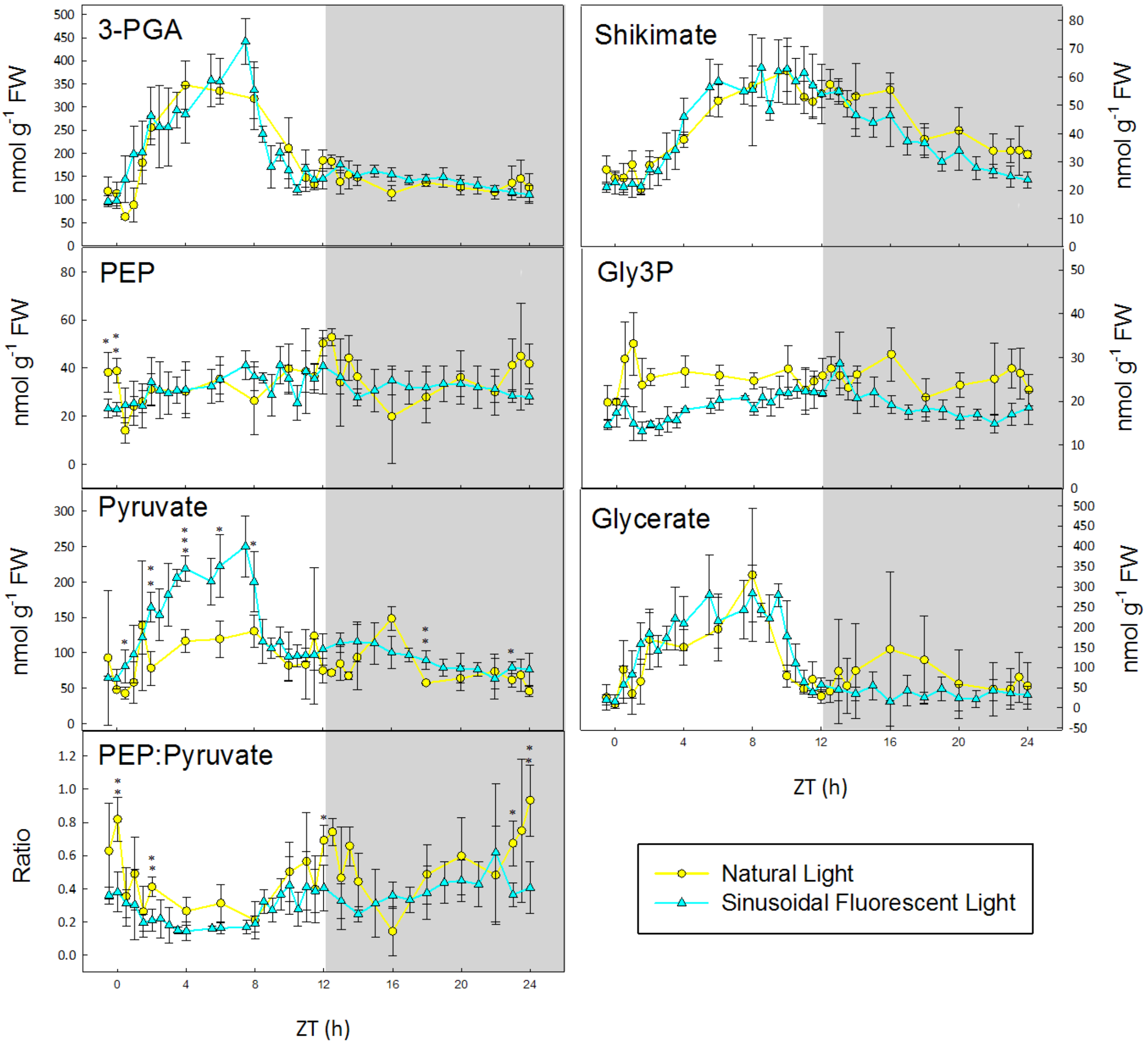

Supplemental Figure S10. (c) Glycolytic intermediates and related metabolites.

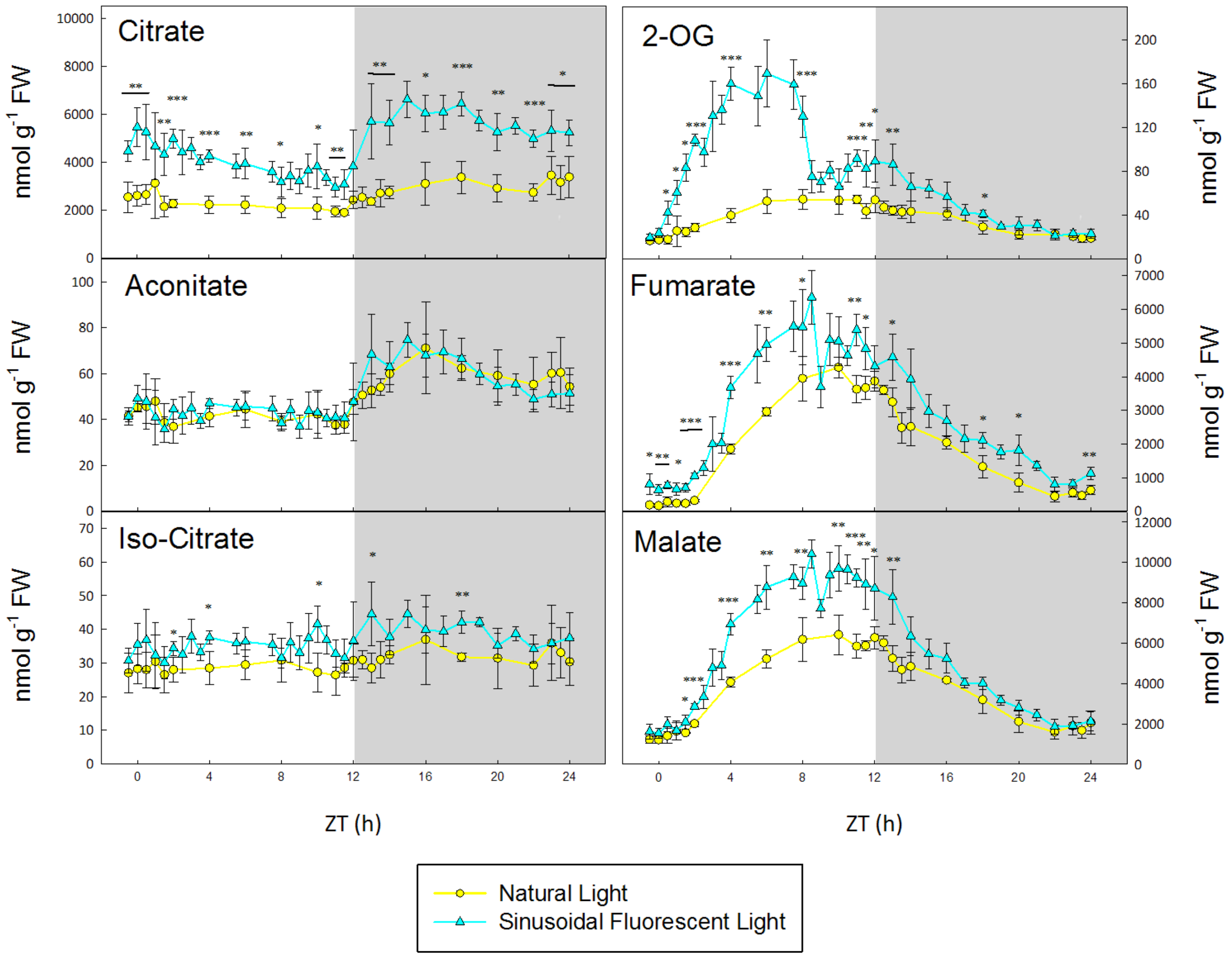

Supplemental Figure S10. (d) TCA cycle intermediates.

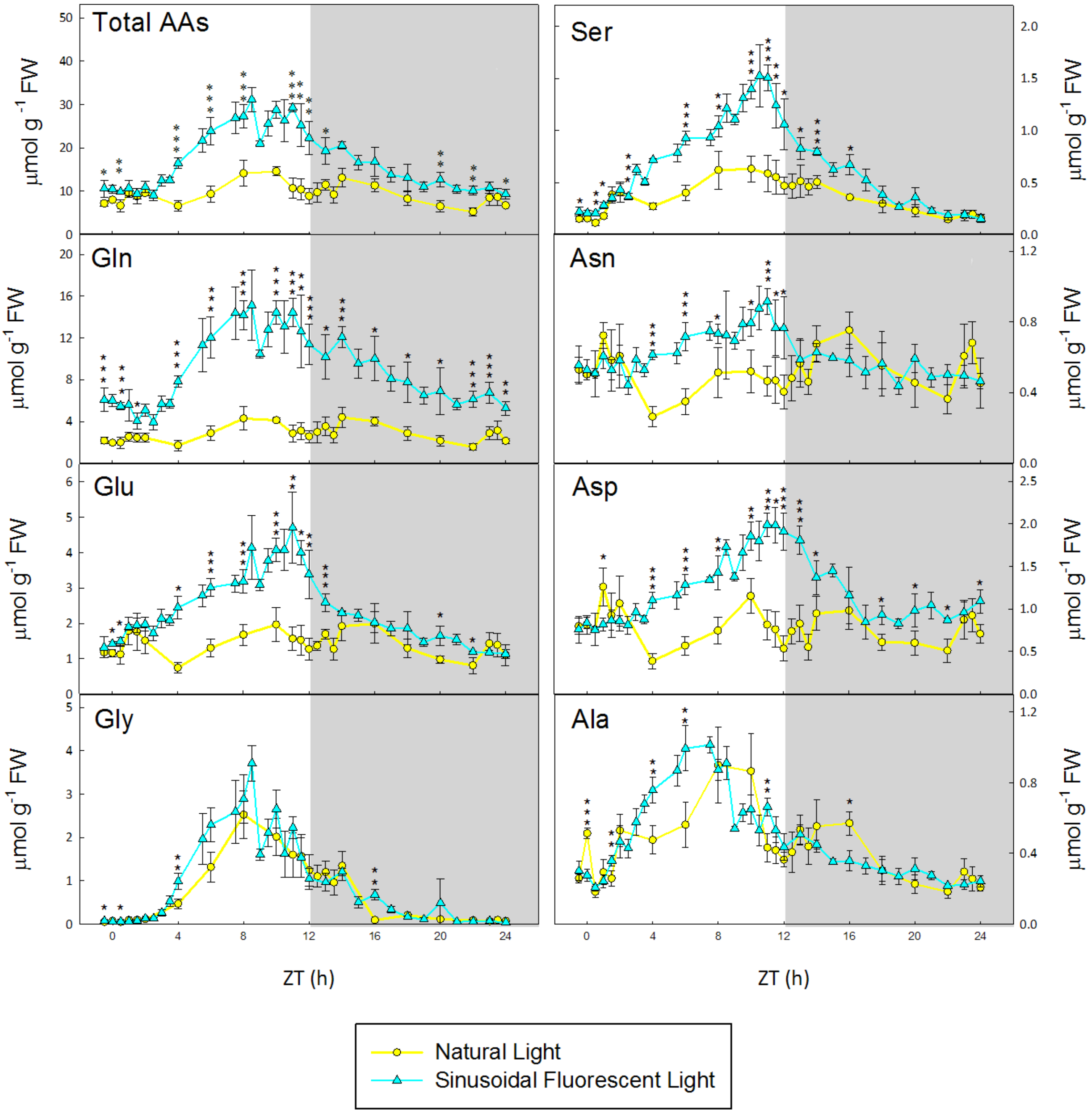

Supplemental Figure S10. (e) Total and major amino acids.

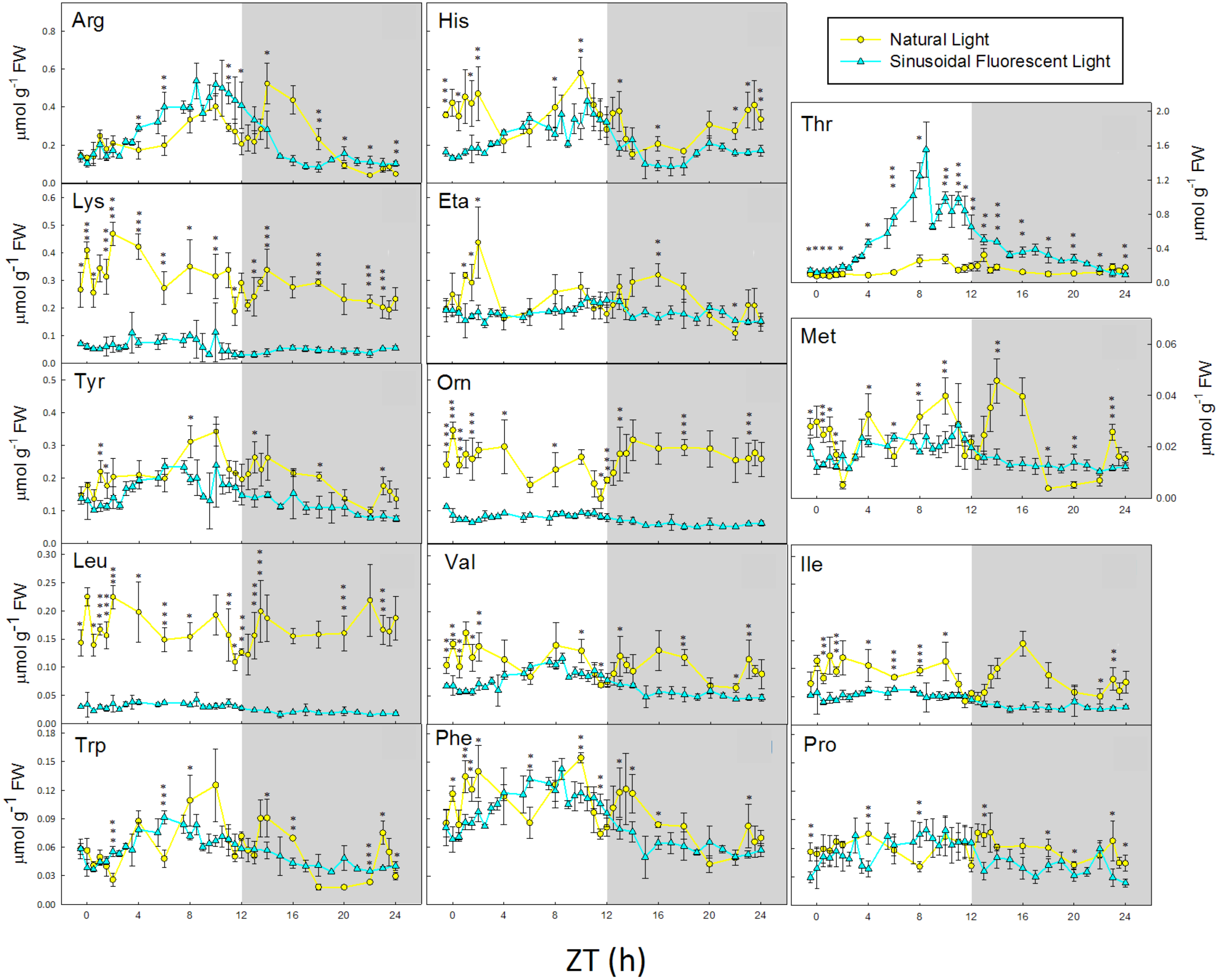

Supplemental Figure S10. (f) Minor amino acids. Eta, ethanolamine; Orn, ornithine

Supplemental Figure S10. Diurnal profiles of metabolites in Arabidopsis plants growing in natural or fluorescent light with a 12-h photoperiod at DLI 12. *Arabidopsis thaliana* Col-0 plants were grown in a naturally illuminated greenhouse around the vernal equinox in 2015 (○) and in a controlled environment chamber with a 12-h photoperiod and DLI of 12 mol·m^{-2}·d^{-1}. Artificial illumination was provided by white fluorescent tubes with a sinusoidal light profile (△). Rosettes were harvested from 4-week-old plants throughout a 24-h diurnal cycle for analysis of: (a) starch and sugars; (b) sugar phosphates and nucleotide sugars; (c) glycolytic intermediates and related metabolite; (d) TCA cycle intermediates; (e) total and major amino acids; and (f) minor amino acids. Data are mean ± SD (*n*=4). ZT, zeitgeber time (hours after dawn).
